# Radiomics in Renal Cell Carcinoma—A Systematic Review and Meta-Analysis

**DOI:** 10.3390/cancers13061348

**Published:** 2021-03-17

**Authors:** Julia Mühlbauer, Luisa Egen, Karl-Friedrich Kowalewski, Maurizio Grilli, Margarete T. Walach, Niklas Westhoff, Philipp Nuhn, Fabian C. Laqua, Bettina Baessler, Maximilian C. Kriegmair

**Affiliations:** 1 Department of Urology and Urological Surgery, University Medical Center Mannheim, University of Heidelberg, Theodor-Kutzer-Ufer 1-3, 68167 Mannheim, Germany; julia.muehlbauer@umm.de (J.M.); luisa.egen@umm.de (L.E.); karl-friedrich.kowalewski@umm.de (K.-F.K.); margarete.walach@umm.de (M.T.W.); niklas.westhoff@umm.de (N.W.); philipp.nuhn@umm.de (P.N.); 2 Library of the Medical Faculty Mannheim of the University of Heidelberg, University Medical Center Mannheim, Theodor-Kutzer-Ufer 1-3, 68167 Mannheim, Germany; maurizio.grilli@medma.uni-heidelberg.de; 3 Institute of Diagnostic and Interventional Radiology, University Hospital Zurich, University of Zurich, Raemistrasse 100, 8091 Zurich, Switzerland; fabianchristopher.laqua@usz.ch (F.C.L.); bettina.baessler@usz.ch (B.B.)

**Keywords:** renal cell carcinoma, computed tomography, magnetic resonance imaging, machine learning, radiomics

## Abstract

**Simple Summary:**

Radiomics may answer questions where the conventional interpretation of medical imaging has limitations. The aim of our systematic review and meta-analysis was to assess the (current) status of evidence in the application of radiomics in the field of renal masses. We focused on its role in diagnosis, sub-entity discrimination and treatment response assessment in renal cell carcinoma (RCC) and benign renal masses. Our quantitative synthesis showed promising results in discrimination of tumor dignity, nevertheless, the value added to human assessment remains unclear and should be the focus of future research. Furthermore, the benefit regarding treatment response assessment remains unclear as well, since the existing studies are investigating already abandoned systemic therapies (ST), which no longer represent the current “reference” standard. Open science could enable to establish technical and clinical validity of radiomic signatures prior to the incorporation of radiomics into everyday clinical practice.

**Abstract:**

Radiomics may increase the diagnostic accuracy of medical imaging for localized and metastatic RCC (mRCC). A systematic review and meta-analysis was performed. Doing so, we comprehensively searched literature databases until May 2020. Studies investigating the diagnostic value of radiomics in differentiation of localized renal tumors and assessment of treatment response to ST in mRCC were included and assessed with respect to their quality using the radiomics quality score (RQS). A total of 113 out of 1098 identified studies met the criteria and were included in qualitative synthesis. Median RQS of all studies was 13.9% (5.0 points, IQR 0.25–7.0 points), and RQS increased over time. Thirty studies were included into the quantitative synthesis: For distinguishing angiomyolipoma, oncocytoma or unspecified benign tumors from RCC, the random effects model showed a log odds ratio (OR) of 2.89 (95%-CI 2.40–3.39, *p* < 0.001), 3.08 (95%-CI 2.09–4.06, *p* < 0.001) and 3.57 (95%-CI 2.69–4.45, *p* < 0.001), respectively. For the general discrimination of benign tumors from RCC log OR was 3.17 (95%-CI 2.73–3.62, *p* < 0.001). Inhomogeneity of the available studies assessing treatment response in mRCC prevented any meaningful meta-analysis. The application of radiomics seems promising for discrimination of renal tumor dignity. Shared data and open science may assist in improving reproducibility of future studies.

## 1. Introduction

The concept of radiomics is based on the assumption that data from biomedical image methods such as computed tomography (CT), positron emission tomography (PET), or magnetic resonance imaging (MRI) contain information of disease- and patient-specific processes that are imperceptible to the human eye [1,2]. Through mathematical extraction of the spatial distribution of signal-intensities and pixel-interrelationships, radiomics may quantify this hidden textural information in a tissue [3,4]. Especially in the field of oncology, the number of studies reporting the application of radiomics to medical imaging data in order to classify a disease type or stage or to predict patient outcome has risen exponentially over the past few years.

This rise includes imaging of renal cell carcinoma (RCC), which represents a common urological malignancy. According to the US National Cancer Institute, 74,000 new cases are diagnosed annually, and the incidence is currently increasing mainly due to the wide use of advanced imaging modalities which incidentally detect renal tumors [5]. Currently, there are two important challenges of traditional radiological imaging in RCC: (i) The limited accuracy in predicting dignity of small renal tumors. Remarkably, up to 30% of resected tumors reveal a benign histology in pathological evaluation after partial or radical nephrectomy [6]. And (ii), the complex assessment of treatment response to systemic therapy (ST) in patients with metastatic RCC (mRCC), where various ST options are available. Especially for immunomodulatory ST, which may result in imaging pseudo progression, current standards of therapy response assessment based on the size of measurable lesions are reaching their limits [7].

Consequently, the recent application of radiomics in RCC has predominately focused on two main goals: (i) to improve the accuracy of preoperative, non-invasive histologic subtyping of small renal tumors in order to improve treatment decision between active treatment and surveillance, and (ii) to optimize and objectify the assessment of therapy response in patients with mRCC under ST in order to prevent a possible wrong therapy change.

A former systematic review and meta-analysis by Ursprung et al. found, that radiomics might be a promising tool to discriminate angiomyolipoma (AML) from RCC [8]. However, the work limited its focus on discriminating AML from RCC and the value of radiomics in the differentiation of other benign tumors-in particular oncocytoma-remained unclear. At this point, an improved diagnostic accuracy would be highly anticipated in clinical practice. A prospective proof-of-concept study showed promising results for characterization of treatment-related changes in multiple radiomics features after ST in integrated PET/MRI [9].

Hence, the aim of our systematic review and meta-analysis was to assess the current evidence for the application of radiomics to renal masses, with a special focus on non-invasive classification of dignity and assessment of treatment response.

## 2. Materials and Methods

This review follows the Cochrane Handbook of Systematic Reviews and Interventions and is in concordance of the AMSTAR-2 criteria and the PRISMA guidelines [10,11,12]. It was prospectively registered on PROSPERO (ID: CRD42020221345).

### 2.1. Sources

A comprehensive database search including MEDLINE (via PubMed), Web of Science, CENTRAL, Cinahl, Current Contests Medicine (CCMed, via LIVIVO) and ClinicalTrials.gov (accessed on 15 February 2021) was conducted by a specialized librarian of the University of Heidelberg (M.G.) in May 2020. No time frame or language restrictions were applied. In addition, the reference lists of the included studies were searched for studies that could potentially also be included and the authors were contacted if necessary.

### 2.2. Inclusion and Exclusion Criteria

All single studies, comparative studies and original studies on humans, which met the following PICO criteria, were included:

- P (patients): Patients with benign or malign renal tumors;
- I (interventions): Radiomics or texture analysis;
- C (comparison): CT or MRI;
- O (outcome): Histologic subtyping (including differentiation of different RCC subtypes, differentiation and/or analysis of any benign and/or malign renal tumors, tumor grading, and any mutation analyses) and treatment response assessment.

We defined treatment response assessment as the prediction of a therapy response including prediction of survival or as the assessment of an existing response under ST including the correlation with survival. Reviews and case studies were excluded

### 2.3. Search Terms

The full search terms can be found in Table S1.

### 2.4. Study Selection

Two reviewers (J.M. and L.E.) independently screened titles and abstracts. Consecutively, the same two reviewers evaluated the full texts of eligible studies, verifying whether the inclusion criteria were met. Disagreements were solved by consensus or a third reviewer (B.B.). 

### 2.5. Quality Assessment

Quality assessment of all included studies was recorded using the radiomics quality score (RQS), which is a radiomics-specific quality assessment tool [1]. It is made of a total of 16 criteria, for each of which a certain number of points can be achieved. The respective number of points corresponds to the importance regarding the methodological quality of a study [1]. The score ranges from -8 to +36 points, with a score of −8 to 0 points corresponding to 0% and 36 points corresponding 100% [13]. For details, see Table 1. The data of included studies were extracted by two reviewers (J.M. and L.E.) independently. Consecutively, the mean of the two evaluations was then calculated for each study to provide the final RQS. Furthermore, the average value for the respective criteria was given for the entirety of all studies, again using the mean value of both reviewers for each respective criterion. 

### 2.6. Meta-Analysis

Two meta-analyses were planned within the included studies:

- a meta-analysis of all studies investigating the use of radiomics to compare benign versus malign renal tumors;
- a meta-analysis of all studies investigating the use of radiomics for treatment response assessment of metastatic RCC with any ST.

Data of all included studies were extracted by two reviewers (J.M. and L.E.) independently. Disagreement was solved by consensus or a third reviewer, if this was necessary (B.B.). Only studies, from which a two-by-two contingency table could be extracted or reconstructed were included. If multiple models were reported in a study, only the one with the highest area under the curve (AUC) was extracted. If no AUC was provided, the model with the highest Youden’s Index was chosen. Models deriving from augmented data were excluded. In case of multiple publications deriving from one study, only the manuscript with better methodological quality according to the RQS was included. 

### 2.7. Statistical Analysis

Random effects meta-analyses were performed using the Mantel-Haenszel model and reported as log odds ratio (OR). Forest plots were used for visualization of the results. Subgroup analyses stratified by different histologic subtypes (AML versus RCC, oncocytoma versus RCC, benign tumors not further specified versus RCC) were performed to evaluate whether findings can be applied across the different subtypes and the entirety of all benign vs. malignant tumors. Due to the small number of included studies, no differentiation between the administered therapies could be made for the subgroup analysis of treatment response assessment. Forest plots were designed to provide subgroup and overall analysis at the same time for each outcome.

The heterogeneity of studies was assessed using the I^2^ index. An I^2^ value of 0–25% represents insignificant heterogeneity, >25–50% low heterogeneity, >50–75% moderate heterogeneity, and >75% high heterogeneity [14]. A *p* value of ≤0.05 was considered as statistically significant. We did not adjust for multiple testing in concordance with earlier arguments for this approach [15]. All analyses were performed using R (R Core Team (2019). R: A language and environment for statistical computing. R Foundation for Statistical Computing, Vienna, Austria) following an a priori specified statistical analysis plan.

## 3. Results

### 3.1. Included Studies

**Figure 1:**
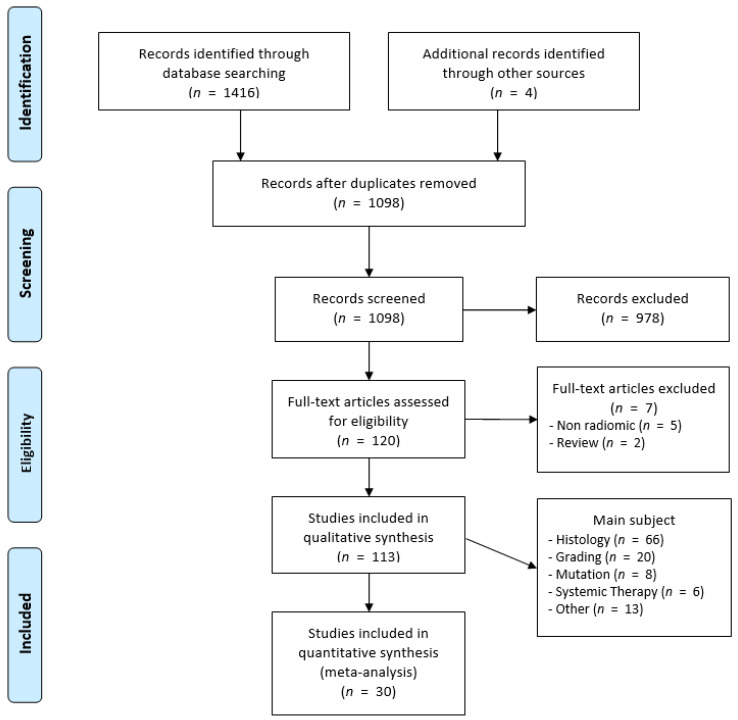
PRISMA Flow-chart of included studies.

Figure 1 shows the PRISMA flow-chart of the included studies of this systematic review and meta-analysis. Table S2 summarizes the characteristics and research questions of the included studies. In the 109 studies for which the number of patients was available, a total of 12,054 patients were included. In four studies, only the number of investigated tumors was given. Most studies investigated the use of radiomics using CT imaging (*n* = 84), less than a quarter based on magnetic resonance (MR) imaging (*n* = 23), and two based on a combination of CT and MR imaging. Another three studies investigated the use of radiomics on PET MR images and one pilot study investigated its use on ^18^F-fluorodeoxyglucose positron emission tomography (FDG PET) images.

**Figure 2:**
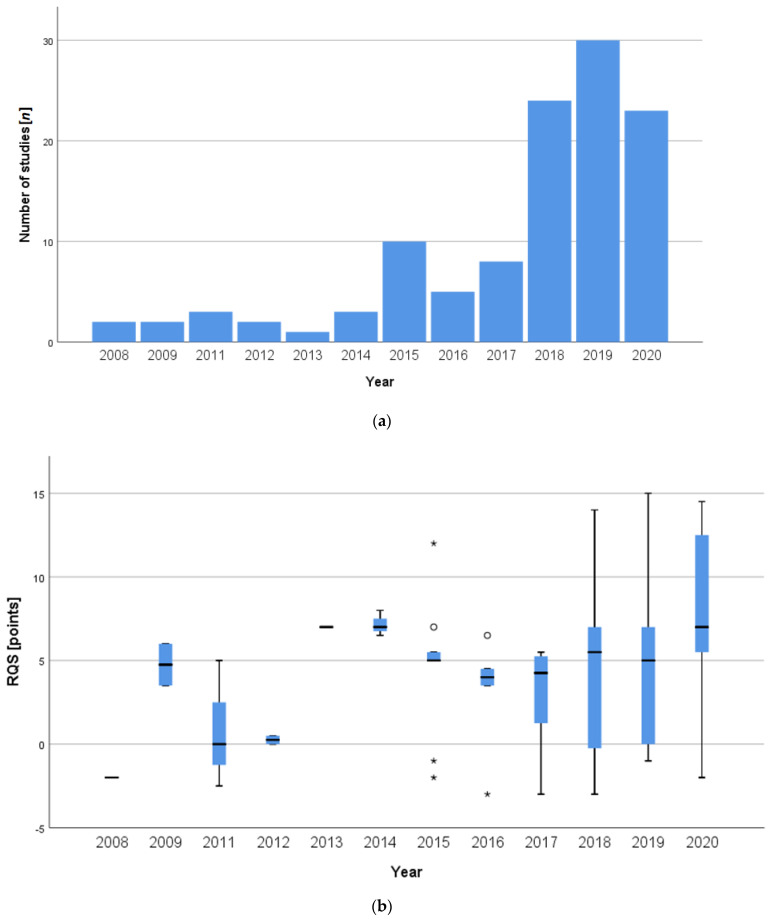
Number (**a**) and box-and-whisker plots (**b**) of the studies over time. In (**b**), the outliers are represented by circles and the extremes by asterisks.

Over the past few years, the number of studies investigating the use of radiomics in RCC has increased continuously. In particular, there has been an increase in the number of studies carried out in the last two years: 47% (*n* = 53) of all included studies were published between 2019 and 2020. Another quarter of all included studies were published in the two previous years (2017 and 2018, *n* = 32), which roughly corresponds to the number of all studies from previous years (Figure 2a).

### 3.2. Quality Assessment

Table 1 shows the average score for each criterion of the RQS of all included studies. The respective ratings for each study are shown in Table S2. The mean RQS score of all included studies was 13.6% (4.9 points, standard deviation (SD) ± 4.60 points), the median score was 13.9% (5.0 points, inter-quartile range (IQR) 0.25–7.0 points) and the range was 0–41.7% (−3.0 to 15.0 points). 

Over time, an increase in the quality according to the RQS of the included studies could be observed (Figure 2b). In 2020 in particular, the RQS was better than in previous years with a mean RQS of 21.6% (7.76 points, SD ± 4.97 points) and a median score of 19.4% (7.0 points, IQR 5.0–13.0 points). The respective ratings over the years are shown in Table S3.

### 3.3. Differentiation of Benign and Malign Renal Tumors

Of the 66 studies which dealt with the prediction of histological subtypes of renal tumors, 52 investigated the prediction of the dignity of renal masses, of which 30 could be included in the quantitative analysis. The most common exclusion criterion was the insufficient disclosure of the results (*n* = 18), which prevented the possibility to extract a two-by-two contingency table. Two of the studies used data augmentation with generation of new data through transformation of existing cases and therefore had to be excluded. Another two studies had to be excluded because multiple publications derived from one study. Table S4 lists further details on the exclusion criteria. Furthermore, Table S4 provides an overview of all 52 studies investigating the prediction of the dignity of renal masses including the respective research question, a brief summary of the used methods, the main results and the used reference for comparison with the use of radiomics for each study. All studies used the histologically confirmed diagnosis for the respective tumor subtype as the gold reference standard. In most cases (*n* = 31), the histological diagnosis was based on the surgically removed tumor by partial or radical nephrectomy. However, some studies (*n* = 21) did not clarify whether the histology came from a biopsy or from the surgically removed tumor. None of the studies indicated that the histological result was only verified by biopsy.

**Figure 3:**
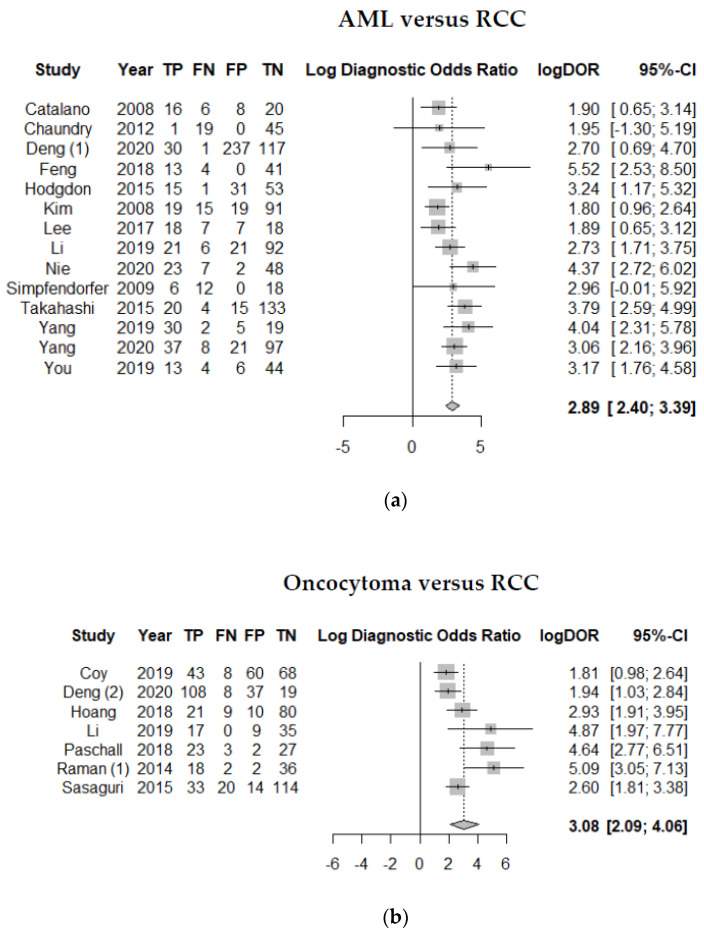

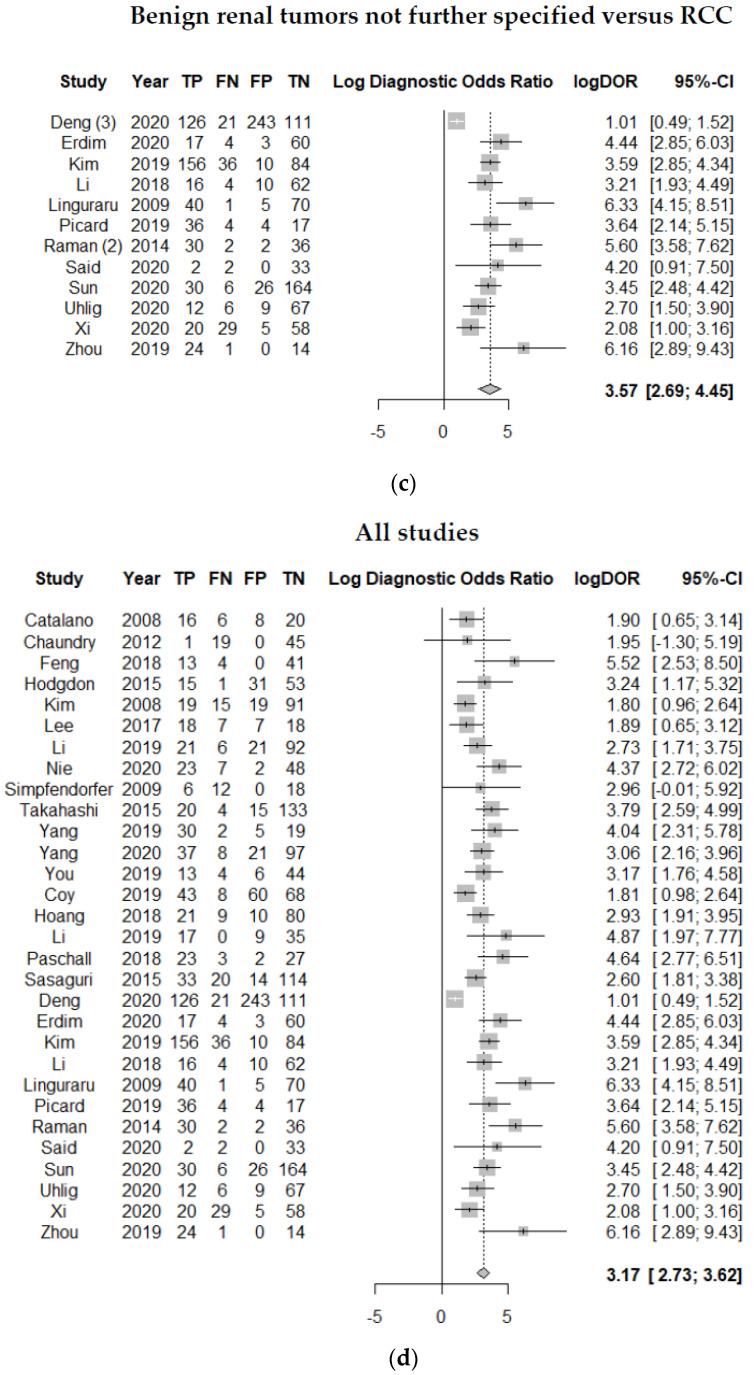
Forest plot of the effect size calculated as log odds ratio for studies investigating (**a**) AML versus RCC, (**b**) oncocytomas versus RCC, (**c**) benign tumors not further specified versus RCC, and (**d**) the sum of all studies. TP: number of AML (**a**), oncocytomas (**b**) or benign renal tumors (**c**) correctly diagnosed; FN: number of AML (**a**), oncocytomas (**b**) or benign renal tumors (**c**) incorrectly diagnosed as RCC; FP: number of RCC incorrectly diagnosed as AML (**a**), oncocytomas (**b**) or benign renal tumors (**c**); TN: number of RCC correctly diagnosed.

Within the 30 included studies, the discrimination of AML and RCC was investigated in 14, the discrimination of oncocytoma and RCC in seven, and the discrimination of unspecified benign renal tumors and RCC in 12 of the studies. Two of the studies (Deng et al. [16] and Raman et al. [17]) investigated the discrimination of RCC from various sub-entities of benign tumors, which explains the multiple listing of these studies in the respective analyses. Figure 3 shows the forest plot of the effect size calculated as log OR for studies investigating AML versus RCC (a), oncocytoma versus RCC (b), benign tumors not further specified versus RCC (c), and the sum of all studies (d).

For differentiating AML versus RCC, the random effects model showed a log OR of 2.89 (95%-CI 2.40–3.39, *p* < 0.001) with low heterogeneity of the studies of I^2^ = 40.5% (95%-CI 0.0–68.4, *p* = 0.058). For the differentiation of oncocytoma versus RCC, log OR was 3.08 (95%-CI 2.09–4.06, *p* < 0.001) with moderate heterogeneity of I^2^ = 67.7% (95%-CI 28.2–85.4, *p* = 0.005), and for the differentiation of benign tumors not further specified versus RCC, log OR was 3.57 (95%-CI 2.69–4.45, *p* < 0.001) with high heterogeneity of 86.3% (95%-CI 77.9–91.6, *p* < 0.001). For the overall analysis of all the above-mentioned studies investigating the differentiation of benign versus malign renal tumors, the random effects model showed a log OR 3.17 (95%-CI 2.73–3.62, *p* < 0.0001) with moderate heterogeneity of 74.6% (95%-CI 63.7–82.2, *p* < 0.001).

### 3.4. Treatment Response Assessment

Overall, six studies were identified which assessed response to ST and prediction of treatment response using radiomics. Table S5 summarizes the characteristics and investigated features of the respective studies. 

Due to the large inhomogeneity especially concerning regions of interest (ROI) and endpoints of the studies, no sufficient data could be extracted that would have enabled a meaningful meta-analysis.

## 4. Discussion

The use of radiomics in oncological imaging for different tumor entities has become increasingly popular in recent years with the overall goal to improve clinical decision-making [1]. The high rate of benign renal tumors diagnosed at pathological evaluation after surgery as well as the increasing complexity of treatment response assessment has also led to a growing interest in using radiomics in the field of renal tumor imaging. A former review including studies until Oct. 2018 found that it might be a promising tool to discriminate AML from RCC, but it also showed that the studies that existed up to this point were of poor quality and therefore must be interpreted with caution [8]. Furthermore, the differentiation of other benign tumors than AML and RCC as well as the possibility of therapy response assessment was not investigated [8].

### 4.1. Quality Assessment

In our systematic review, we observed only a slight increase of the quality of radiomics studies in recent years with best quality scores in 2020 (median RQS 19.4% (7.0 points, IQR 5.0–13.0 points)). However, the overall quality was still low (median RQS 13.9% (5.0 points, IQR 0.25–7.0 points)). The main reasons for the low quality of the studies were the deficiency in using feature reduction, the lack of internal and external validation data as well as the lack of use of open science and data. These results are in line with the findings by Ursprung et al. who found a ratio between analysed features and patients ranging from 25 to 240 times more features than patients with only 51% of the included studies in their analysis using feature reduction methods [8]. Furthermore, they also reported a low rate of only 5% of studies who performed validation and found that methodological aspects of the radiomics studies in this field might further increase the risk of bias [8]. These methodological aspects contain the patient selection in particular and the timing of index and reference tests [8]. The poor quality of reporting prediction model studies is a known problem, which amongst others led to the TRIPOD (Transparent Reporting of a multivariable prediction model for Individual Prognosis Or Diagnoses) initiative [13,18]. A set of recommendations was established for the reporting of the studies assessing prediction models to minimize bias and to increase their clinical usefulness [18]. A relatively large number of radiomics studies are pilot or feasibility studies, but they do not provide open data or the codes [17,19,20]. This prevents validation analyses to confirm the respective results. Code and data sharing (“Open Science”) is likely to improve reproducibility in subsequent studies. Especially, the application of deep learning methods for image segmentation will not only highly reduce the time needed for manual segmentation but minimizes potential bias and variance induced by human readers [21].

Proceedings in standardization and homogenization are likely to reduce bias even further following the overall goal to evaluate the real added value of radiomics. In addition, cost-effectiveness analyses are necessary regarding future investigations. These play a crucial role in understanding the clinical and economic consequences of this technique, as well as its possible future integration into everyday clinical practice [22]. 

### 4.2. Classification of Dignity of Renal Tumors

Our meta-analysis of all studies investigating the differentiation of benign versus malign renal tumors showed promising results in the overall (log OR 3.17, 95%-CI 2.73–3.62, *p* < 0.0001) and the subgroup analyses. Nevertheless, these results must be considered with care.

First, the inconsistency of the use of radiomics in clinical practice should be considered. This inconsistency is represented by methodological differences between the included studies in our meta-analysis as well as the inconsistent and partly insufficient disclosure of the results in the respective studies. As a result, a large part of the studies investigating the dignity of renal masses (22 out of 52 studies) could unfortunately not be included in the meta-analysis, which could possibly have biased our results.

Second, given the overall low quality of the analysed studies when it comes to the RQS as well as the well-known vulnerability of radiomics to a large number of technical and non-technical factors, machine learning models are highly prone to overfitting due to the usually high number of radiomics features, considerably exceeding the number of patients included in the study [23]. Algorithms performing complex calculations with radiomics data might easily be confounded by the tiniest amount of noise, and without adhering to strong quality control of study design and especially statistical analysis, it is very likely that the results of our meta-analysis are over-optimistic. 

Third, it should be noted that all studies included in the meta-analysis used histopathological findings as the reference standard, which was based on the surgically removed tumor in most cases. However, since the use of radiomics is crucial for clinical decision-making in the preoperative setting, the comparison with alternative diagnostic options, which are available as reference standards in the preoperative context, would be a more meaningful comparison for assessing the real added value of radiomics. Such diagnostic options are, on the one hand, a histologically confirmed diagnosis based on renal biopsies and, on the other hand, human imaging assessment. In comparison to human assessment, the results of Uhlig et al. and Sun et al. suggest, that the use of radiomics and machine learning could provide an added value to differentiate benign and malign renal lesions [24,25]. Picard et al. and Said et al. furthermore found that the combination of qualitative and quantitative image analyses seems to be a promising approach [26,27]. In contrast, Takahashi et al. did not find a significant benefit of radiomics compared to human assessment [28].

In summary, although the majority of the results indicate that radiomics could be a valuable tool to differentiate benign and malignant renal tumors and that it might bring an added value compared to and in combination with human assessment, the following should be considered for future studies in this context: To confirm the added value or even a superior diagnostic or prognostic performance of radiomics as compared to other available diagnostic options in the preoperative context, human assessment and the comparison to histologically confirmed diagnoses based on renal biopsies should be considered as a reference standard for future studies in addition to histopathological findings based on surgically removed tumors. Furthermore, adherence to rigorous quality standards and complete as well as consistent disclosure of the results should be ensured.

Ultimately, prospective preferably (cluster) randomized controlled trials would need to be conducted for evaluation of promising models comparing the outcomes following treatment decisions based on those predictions to the current human benchmark or other preoperative available diagnostic options. However, the results of our meta-analysis suggest, that especially model stability is not yet suitable to allow for such a step in this research area.

### 4.3. Treatment Response Assessment

All available studies investigating the use of radiomics for therapy response assessment for which the information regarding the respective treatment was available included patients receiving different tyrosine kinase inhibitors (TKI) [9,29,30,31,32]. For patients receiving sunitinib, Antunes et al. found in their proof-of-concept study including two patients, that radiomics might be a powerful tool to characterize treatment related changes in integrated PET/MRI [9], highlighting the potential of additional functional/hybrid imaging in radiomics as well as in response assessment. Furthermore, the results of Bharwani et al. and Haider et al. suggest that changes in different histogram parameters in MRI and CT might be associated with the overall and progression-free survival of patients receiving sunitinib [29,32]. Although these results seem promising, the inhomogeneity of all the studies investigating the therapy response assessment using radiomics unfortunately did not enable a meaningful meta-analysis for these studies. The main problem was the diversity of investigated endpoints. Of the three studies for which the data for the therapy response assessment could be extracted, the change in different radiomics features was investigated. This made a direct comparison not useful. Furthermore, the diversity of the analysed ROIs and of the included ST, as well as the small number of included patients also counteracted a meaningful meta-analysis for these studies. Moreover, it should be noted that none of the studies investigating the use of radiomics to assess therapy response included patients with immunomodulatory therapies, which represent the current reference standard in ST for mRCC [33]. With regard to future investigations for the assessment of therapy response using radiomics, the steadily increasing number of available therapeutic options will be a hurdle. However, it does not seem useful to integrate the selection of relevant radiomics features into the study-protocols of prospective trials investigating the effectiveness of new ST [1]. Standardised and optimized study protocols carry the risk that the feature selection depends more on the imaging process rather than the pathology [34]. Rather, the goal should be to share the data from such studies so that the biological correlation of already established radiomic signatures can be performed [34].

### 4.4. Limitations

Several limitations of this review merit consideration. First, the heterogeneity of the studies included in the quantitative synthesis must be mentioned. This relates not only to the control groups used (different subtypes of RCC or no classification of RCC subtype), but also to the assessed imaging (MRI and CT), the imaging protocols and the deriving phases of the respective imaging from which the features were extracted. In addition, newly investigated image modalities like the use of single-photon emission computed tomography (SPECT)/CT and the use of quantitative SPECT/CT reconstruction methods in the field of renal tumor diagnostic management, which showed promising results in recently published studies, were not considered in this review [35,36,37,38]. Furthermore, the included studies differed in terms of the methodology of the used image reconstruction, feature extraction, and the algorithms used. Due to the inhomogeneity of the reported results, only 30 of the 52 available studies assessing the differentiation of benign and malign renal tumors could be included in the quantitative analysis. Beyond that, no meta-analysis regarding the assessment of treatment response using radiomics could be carried out due to the heterogeneity of the methods and reported results of the available studies. Nevertheless, the results of the quantitative synthesis provide an overview of the benefit that the use of radiomics might have. 

## 5. Conclusions

Our review shows that there has been an increasing number of studies investigating the use of radiomics for RCC, especially in recent years. Even if the quality of the studies improved over time, the RQS in recent years was still quite low. Our quantitative synthesis showed promising results regarding the differentiation between benign and malign renal tumors using radiomics. However, the results are likely over-optimistic and must be considered with care and hence the added value compared to human assessment accuracy remains unclear. Human assessment should be considered as a reference standard for future studies in addition to histopathological findings and adherence to rigorous quality standards should be ensured. Furthermore, the benefit regarding therapy response assessment remains unclear as the existing studies are investigating ST which no longer correspond to the current reference standard. Data sharing of prospective trials investigating new ST might enable to perform biological correlation of already established radiomic signatures.

This review reveals that there are still hurdles to overcome before the added value of radiomics in the diagnostic of RCC can be ascertained. Above all, the weak points are the heterogeneity and missing standardization of the available studies and the lack of evaluation of the benefit against current standards, which represent decisive milestones before the use of radiomics will find its way into everyday clinical practice. By highlighting these challenges, this review can improve the planning of subsequent studies in this context. 

## Figures and Tables

**Table 1:** RQS and average score for the included studies.

Criteria		Points	Average Score
1	Image protocol quality	+1 if protocols are well-documented +1 if public protocol is used	0.65
2	Multiple segmentations	+1 if multiple segmentations are carried out (i.e., different physicians/algorithms/software)	0.53
3	Phantom study	+1 if phantom study is used on all scanners	0.00
4	Multiple time points	+1 if images are collected at additional time points	0.07
5	Feature reduction or adjustment for multiple testing	−3 if neither measure is implemented +3 if either measure is implemented	0.69
6	Multivariable analysis with non-radiomics features	+1 if multivariable analysis with non-radiomics features is carried out	0.12
7	Biological correlates	+1 if phenotypic differences are demonstrated	0.96
8	Cut-of-analyses	+1 if risk groups are determined by either the median, a previously published cut-off or if a continuous risk variable is reported	0.09
9	Discrimination statistics	+1 if a discrimination statistic and its statistical significance is reported (i.e., ROC curve, AUC) +1 if a resampling method technique is also applied (i.e., bootstrapping, cross-validation)	1.21
10	Calibration statistics	+1 if a calibration statistic and its statistical significance is reported (i.e. Calibration-in-the-large/slope) +1 if a resampling method technique is also applied (i.e., bootstrapping, cross-validation)	0.02
11	Prospective	+7 for prospective validation of a radiomics signature in an appropriate trial	0.46
12	Validation	−5 if validation is missing +2 if validation is based on a dataset from the same institute +3 if validation is based on a dataset from another institute +4 if validation is based on two datasets from two institutes +4 if study validates a previously published signature +5 of validation is based in three or more datasets from distinct institutes	−3.88
13	Gold standard	+2 if comparison to the current gold standard is carried out	1.91
14	Potential clinical utility	+2 if a potential application in a clinical setting is reported	2.00
15	Cost-effectiveness analysis	+1 if the cost-effectiveness of the clinical application is reported	0.00
16	Open science and data	+1 if scans are open source +1 if region of interest (ROI) segmentations are open source +1 if code is open source +1 if representative segmentations and features are open source	0.16

## Data Availability

The data presented in this study are available on request from the corresponding author.

## Supplementary Materials

The following are available online at https://www.mdpi.com/2072-6694/13/6/1348/s1, Table S1: Search terms, Table S2: Summary of studies included in the qualitative synthesis (*n* = 113), Table S3: RQS over time, Table S4: Summary of studies investigating the dignity of renal masses (*n* = 52) and reasons for exclusion from the meta-analysis, Table S5: Studies investigating treatment response assessment using radiomics (*n* = 6).
