# Supplementary material for: Radiomics in Renal Cell Carcinoma—A Systematic Review and Meta-Analysis
Source: Cancers (Basel). 2021 Mar 17;13(6):1348. doi: 10.3390/cancers13061348 (PMC8002585; doi:10.3390/cancers13061348)
Supplement: Supplementary file 1 [file cancers-13-01348-s001.pdf]

Supplementary Material

Radiomics in Renal Cell Carcinoma - A Systematic Review and Meta-Analysis

Julia Mühlbauer [1,†], Luisa Egen [1,†], Karl-Friedrich Kowalweski [1], Maurizio Grilli [2], Margarete T. Walach [1], Niklas Westhoff [1], Philipp Nuhn [1], Fabian C. Laqua [3], Bettina Baessler [3,†] and Maximilian C. Kriegmair [1,†,*]

[1] Department of Urology and Urological Surgery, University Medical Center Mennheim, University of Heidelberg Theodor-Kutzer-Ufer 1-3, 68167 Mannheim, Germany; julia.muehlbauer@umm.de (J.M.); Luisa.Egen@umm.de (L.E.); karl-friedrich.kowalewski@umm.de (K.-F.K.); margarete.walach@umm.de (M.T.W.); Niklas.Westhoff@umm.de (N.W.); philipp.nuhn@umm.de (P.N.); maximilian.kriegmair@medma.uni-heidelberg.de (M.C.K.)
[2] Library of Medical Faculty Mannheim of the University of Heidelberg, University Medical Center Mannheim, Theodor-Kutzer-Ufer 1-3, 68167 Mannheim, Germany; maurizio.grilli@medma.uni-heidelberg.de (M.G.)
[3] Institute of Diagnostic and Interventional Radiology, University Hospital Zurich, University of Zurich, Raemistrasse 100, 8091 Zurich, Switzerland; FabianChristopher.laqua@usz.ch (F.C.L.); Bettina.baessler@usz.ch (B.B)
* Correspondence: maximilian.kriegmair@medma.uni-heidelberg.de
† These authors contributed equally to this work.

Supplementary Table S1: Search terms

Database	Hits	Date	Criterion	Search terms
**Pubmed**	**945**	**14.05.2020**		
			**P**	**"Kidney Neoplasms"[Mesh] OR**<br>**"Carcinoma, Renal Cell"[Mesh] OR**<br>**"Oncocytoma, renal"[Supplementary Concept] OR**<br>Kidney Neoplasm*[tiab] OR<br>Renal Neoplasm*[tiab] OR<br>Renal cell neoplasm*[tiab] OR<br>Kidney Cancer*[tiab] OR<br>Renal Cancer*[tiab] OR<br>Renal Cell Cancer*[tiab] OR<br>kidney tumor*[tiab] OR<br>renal tumor*[tiab] OR<br>renal cell tumor*[tiab] OR

				grawitz tumor*[tiab] OR<br>kidney tumour*[tiab] OR<br>renal tumour*[tiab] OR<br>renal cell tumour*[tiab] OR<br>grawitz tumour*[tiab] OR<br>kidney carcinoma*[tiab] OR<br>renal carcinoma*[tiab] OR<br>renal cell carcinoma*[tiab] OR<br>"grawitz carcinoma"[tiab] OR<br>Hypernephroid Carcinoma*[tiab] OR<br>"Cancer of the Kidney"[tiab] OR<br>"Cancer of Kidney"[tiab] OR<br>"Adenocarcinoma of Kidney"[tiab] OR<br>"Tumor of kidney"[tiab] OR<br>"tumour of kidney"[tiab] OR<br>Renal Cell Adenocarcinoma*[tiab] OR<br>Hypernephroma*[tiab] OR<br>Collecting Duct Carcinoma*[tiab] OR<br>Oncocytoma[tiab] OR<br>Angiomyolipoma[tiab]
			**I**	**"Image Processing, Computer-Assisted"[Mesh] OR**<br>Computer Assisted Image*[tiab] OR<br>Image Reconstruction*[tiab] OR<br>radiomics[tiab] OR<br>textur*[tiab] OR
				**"Algorithms"[Mesh] OR**<br>Histogram*[tiab] OR<br>Algorithm*[tiab]
			**C**	**"Positron Emission Tomography Computed Tomography"[Mesh] OR**<br>**"Tomography, X-Ray Computed"[Mesh] OR**<br>"computed tomography"[tiab] OR<br>"Computerized Tomography"[tiab] OR

				"Computed X Ray Tomography"[tiab] OR<br>"Computerized X Ray Tomography"[tiab] OR<br>"Computer Assisted Tomography"[tiab] OR<br>"Computerized Axial Tomography"[tiab] OR<br>"Computer Axial Tomography"[tiab] OR<br>"Computed Axial Tomography"[tiab] OR<br>CT[tiab] OR<br>CAT[tiab] OR
				**"Magnetic Resonance Imaging"[Mesh] OR**<br>"magnetic resonance"[tiab] OR<br>MRI[tiab] OR<br>MR[tiab]
**Cochrane Library**	**27**	**14.05.2020**	**P**	[mh "Kidney Neoplasms"] OR<br>[mh "Carcinoma, Renal Cell"] OR<br>("Kidney Neoplasm*"):ti,ab,kw OR<br>("Renal Neoplasm*"):ti,ab,kw OR<br>("Renal cell neoplasm*"):ti,ab,kw OR<br>("Kidney Cancer*"):ti,ab,kw OR<br>("Renal Cancer*"):ti,ab,kw OR<br>("Renal Cell Cancer*"):ti,ab,kw OR<br>("kidney tumor*"):ti,ab,kw OR<br>("renal tumor*"):ti,ab,kw OR<br>("renal cell tumor*"):ti,ab,kw OR<br>("grawitz tumor*"):ti,ab,kw OR<br>("kidney tumour*"):ti,ab,kw OR<br>("renal tumour*"):ti,ab,kw OR<br>("renal cell tumour*"):ti,ab,kw OR<br>("grawitz tumour*"):ti,ab,kw OR<br>("kidney carcinoma*"):ti,ab,kw OR<br>("renal carcinoma*"):ti,ab,kw OR<br>("renal cell carcinoma*"):ti,ab,kw OR<br>("grawitz carcinoma"):ti,ab,kw OR

				("Hypernephroid Carcinoma*"):ti,ab,kw OR<br>("Cancer of the Kidney"):ti,ab,kw OR<br>("Cancer of Kidney"):ti,ab,kw OR<br>("Adenocarcinoma of Kidney"):ti,ab,kw OR<br>("Tumor of kidney"):ti,ab,kw OR<br>("tumour of kidney"):ti,ab,kw OR<br>("Renal Cell Adenocarcinoma*"):ti,ab,kw OR<br>("Hypernephroma*"):ti,ab,kw OR<br>("Collecting Duct Carcinoma*"):ti,ab,kw OR<br>("Oncocytoma"):ti,ab,kw OR<br>("Angiomyolipoma"):ti,ab,kw
			**I**	[mh "Image Processing, Computer-Assisted"] OR<br>("Computer Assisted Image*"):ti,ab,kw OR<br>("Image Reconstruction*"):ti,ab,kw OR<br>("radiomics"):ti,ab,kw OR<br>("textur*"):ti,ab,kw OR<br>[mh "Algorithms"] OR<br>("Histogram*"):ti,ab,kw OR<br>("Algorithm*"):ti,ab,kw
			**C**	[mh "Positron Emission Tomography Computed Tomography"] OR<br>[mh "Tomography, X-Ray Computed"] OR<br>("computed tomography"):ti,ab,kw OR<br>("Computerized Tomography"):ti,ab,kw OR<br>("Computed X Ray Tomography"):ti,ab,kw OR<br>("Computerized X Ray Tomography"):ti,ab,kw OR<br>("Computer Assisted Tomography"):ti,ab,kw OR<br>("Computerized Axial Tomography"):ti,ab,kw OR<br>("Computer Axial Tomography"):ti,ab,kw OR<br>("Computed Axial Tomography"):ti,ab,kw OR<br>("CT"):ti,ab,kw OR<br>("CAT"):ti,ab,kw OR<br>[mh "Magnetic Resonance Imaging"] OR

				("magnetic resonance"):ti,ab,kw OR<br>("MRI"):ti,ab,kw OR<br>("MR"):ti,ab,kw
**Web of Science Core Collection**	**307**	**14.05.2020**	**P**	"Kidney Neoplasm*" OR<br>"Renal Neoplasm*" OR<br>"Renal cell neoplasm*" OR<br>"Kidney Cancer*" OR<br>"Renal Cancer*" OR<br>"Renal Cell Cancer*" OR<br>"kidney tumor*" OR<br>"renal tumor*" OR<br>"renal cell tumor*" OR<br>"grawitz tumor*" OR<br>"kidney tumour*" OR<br>"renal tumour*" OR<br>"renal cell tumour*" OR<br>"grawitz tumour*" OR<br>"kidney carcinoma*" OR<br>"renal carcinoma*" OR<br>"renal cell carcinoma*" OR<br>"grawitz carcinoma" OR<br>"Hypernephroid Carcinoma*" OR<br>"Cancer of the Kidney" OR<br>"Cancer of Kidney" OR<br>"Adenocarcinoma of Kidney" OR<br>"Tumor of kidney" OR<br>"tumour of kidney" OR<br>"Renal Cell Adenocarcinoma*" OR<br>"Hypernephroma*" OR<br>"Collecting Duct Carcinoma*" OR<br>"Oncocytoma" OR<br>"Angiomyolipoma"

			**I**	"Computer Assisted Image*" OR<br>"Image Reconstruction*" OR<br>"radiomics" OR<br>"textur*" OR<br>"Histogram*" OR<br>"Algorithm*"
			**C**	"computed tomography" OR<br>"Computerized Tomography" OR<br>"Computed X Ray Tomography" OR<br>"Computerized X Ray Tomography" OR<br>"Computer Assisted Tomography" OR<br>"Computerized Axial Tomography" OR<br>"Computer Axial Tomography" OR<br>"Computed Axial Tomography" OR<br>"CT" OR<br>"CAT" OR<br>"magnetic resonance" OR<br>"MRI" OR<br>"MR"
**CINAHL**	**120**	**14.05.2020**	**P**	"Kidney Neoplasm*" OR<br>"Renal Neoplasm*" OR<br>"Renal cell neoplasm*" OR<br>"Kidney Cancer*" OR<br>"Renal Cancer*" OR<br>"Renal Cell Cancer*" OR<br>"kidney tumor*" OR<br>"renal tumor*" OR<br>"renal cell tumor*" OR<br>"grawitz tumor*" OR<br>"kidney tumour*" OR<br>"renal tumour*" OR<br>"renal cell tumour*" OR

				"grawitz tumour*" OR<br>"kidney carcinoma*" OR<br>"renal carcinoma*" OR<br>"renal cell carcinoma*" OR<br>"grawitz carcinoma" OR<br>"Hypernephroid Carcinoma*" OR<br>"Cancer of the Kidney" OR<br>"Cancer of Kidney" OR<br>"Adenocarcinoma of Kidney" OR<br>"Tumor of kidney" OR<br>"tumour of kidney" OR<br>"Renal Cell Adenocarcinoma*" OR<br>"Hypernephroma*" OR<br>"Collecting Duct Carcinoma*" OR<br>"Oncocytoma" OR<br>"Angiomyolipoma"
			**I**	"Computer Assisted Image*" OR<br>"Image Reconstruction*" OR<br>"radiomics" OR<br>"textur*" OR<br>"Histogram*" OR<br>"Algorithm*"
			**C**	"computed tomography" OR<br>"Computerized Tomography" OR<br>"Computed X Ray Tomography" OR<br>"Computerized X Ray Tomography" OR<br>"Computer Assisted Tomography" OR<br>"Computerized Axial Tomography" OR<br>"Computer Axial Tomography" OR<br>"Computed Axial Tomography" OR<br>"CT" OR<br>"CAT" OR

				"magnetic resonance" OR<br>"MRI" OR<br>"MR"
**CCMed**	**1**	**14.05.2020**	**P**	Kidney Neoplasm OR<br>Renal Neoplasm OR<br>Nierentumor
			**I**	Computer Assisted Image OR<br>Image Reconstruction OR<br>radiomics OR<br>textur OR<br>Histogram OR<br>Algorithm
			**C**	computed tomography OR<br>Computerized Tomography OR<br>Computed X Ray Tomography OR<br>Computerized X Ray Tomography OR<br>Computer Assisted Tomography OR<br>Computerized Axial Tomography OR<br>Computer Axial Tomography OR<br>Computed Axial Tomography OR<br>CT OR<br>CAT OR<br>magnetic resonance OR<br>MRI OR<br>MR

**Clinical Trial Gov**<br>**http://www.clinicaltrials.gov/**	**16**	**14.05.2020**	**P**	"Kidney Neoplasm" OR<br>"Renal Neoplasm" OR<br>"Renal Cell Adenocarcinoma" OR<br>"Hypernephroma" OR<br>"Collecting Duct Carcinoma" OR<br>"Oncocytoma" OR<br>"Angiomyolipoma"
			**I**	"Computer Assisted Image" OR<br>"Image Reconstruction" OR<br>"radiomics" OR<br>"textur" OR<br>"Histogram" OR<br>"Algorithm"
			**C**	"computed tomography" OR<br>"Computerized Tomography" OR<br>"Computed X Ray Tomography" OR<br>"Computerized X Ray Tomography" OR<br>"Computer Assisted Tomography" OR<br>"Computerized Axial Tomography" OR<br>"Computer Axial Tomography" OR<br>"Computed Axial Tomography" OR<br>"CT" OR<br>"CAT" OR<br>"magnetic resonance" OR<br>"MRI" OR<br>"MR"

Supplementary Table S2: Summary of studies included in the qualitative synthesis (n = 113)

**Author**	**Year**	**Journal**	**Nr. Pat.**	**Modality**	**Research question**	**RQS [% (points)]**
**Antunes [1]**	2016	*Trans Oncol*	2	PET/MR	Treatment response to antiangiogenic therapy	11.1 (4)
**Bagci [2]**	2013	*PLoS One*	30	18F-FDG-PET	Automated identification of different lesions (metastatic papRCC, cerebellar haemangioblastoma, NSCLC, neurofibroma, lymphomatoid granulomatosis, lung neoplasm, neuroendocrine tumor, soft tissue thoracic mass, nonnecrotizing granulomatous inflammation, RCC with papillary and cystic features, diffuse large B-cell lymphoma, metastatic alveolar soft part sarcoma, SCLC) and prediction of future morphological changes of lesions	19.4 (7)
**Bektas [3]**	2019	*Eur Radiol*	53	CT	Prediction of Fuhrman Grade: High versus low grade RCC	13.9 (5)
**Bharwani [4]**	2014	*Br J Cancer*	20	MRI	Treatment response to antiangiogenic therapy: Treatment-associated texture changes under sunitinib and correlation with OS	22.2 (8)
**Bier [5]**	2018	*PLoS One*	106	CT	Prediction of tumor nuclear grade and prediction of peri- and postoperative complications and blood loss	15.3 (5.5)
**Boos [6]**	2017	*AJR Am J Roentgenol*	19	CT	Treatment response to antiangiogenic therapy: Median versus mean attenuation in the response assessment (RECIST) in patients treated with VEGF TKI	0 (-3)
**Catalano [7]**	2008	*Radiology*	50	CT	Differentiation of AMLwvf from ccRCC	0 (-2)
**Chandarana [8]**	2012	*Radiology*	73	MRI	Differentiation of ccRCC and papRCC	1.4 (0.5)
**Chaudhry [9]**	2012	*AJR Am J Roentgenol*	64	CT	Differentiation of AMLwvf and ccRCC and papRCC	0 (0)
**Chen [10]**	2017	*Abdom Radiol (NY)*	94	CT	Differentiation of ccRCC from renal oncocytoma	15.3 (5.5)
**Chen [11]**	2015	*Springerplus*	61	CT	Differentiation of ccRCC and papRCC	13.9 (5)
**Chen [12]**	2018	*Phys Med Biol*	57	CT	Prediction of the mutation status of ccRCC (VHL, PBRM1 and BAP 1)	4.2 (1.5)
**Coy [13]**	2019	*Abdom Radiol (NY)*	179	CT	Differentiation of ccRCC and oncocytoma	11.1 (4)

**Cui [14]**	2019	*Acta Radiol*	171	CT	Differentiation of AMLwvf from RCC	29.2 (10.5)
**Cui [15]**	2020	*Eur Radiol*	460	CT and MRI	Prediction of Fuhrman Grade: High versus low grade RCC	31.9 (11.5)
**Deng [16]**	2020	*Clin Radiol*	501	CT	Differentiation of RCC and benign renal tumors (AML vs. RCC, Oncocytoma vs. chrRCC, benign vs. malignant)	0 (-1)
**Deng [17]**	2019	*Eur Radiol*	290	CT	Differentiation of ccRCC and papRCC and prediction of Fuhrmann grade: high versus low grade RCC	0 (-1)
**Ding [18]**	2018	*Eur J Radiol*	206	CT	Prediction of Fuhrman Grade: High versus low grade ccRCC	38.9 (14)
**Doshi [19]**	2016	*AJR Am J Roentgenol*	38	MRI	Differentiation of papRCC type 1 and papRCC type 2	9.7 (3.5)
**Dwivedi [20]**	2018	*Urology*	6	MRI	Implementation of a platform for co-localization of in vivo quantitative mpMRI features with ex vivo surgical specimen of renal masses using 3D-printed tumor molds	0 (-2.5)
**Erdim [21]**	2020	*Acad Radiol*	79	CT	Differentiation of benign and malignant renal tumors	19.4 (7)
**Feng [22]**	2018	*Eur Radiol*	58	CT	Differentiation of AMLwvf and RCC (multiple subtypes)	19.4 (7)
**Feng [23]**	2019	*Cancer Imaging*	131	CT	Prediction of Fuhrman Grade: High versus low grade ccRCC	0 (-0.5)
**Feng [24]**	2020	*Front Oncol*	54	CT	Prediction of BRCA1-associated protein 1 mutation status in ccRCC	19.4 (7)
**Gaing [25]**	2015	*Invest Radiol*	44	MRI	Differentiation of ccRCC, papRCC, chrRCC, oncocytoma and AML	33.3 (12)
**Ghosh [26]**	2015	*J Med Imaging (Bellingham)*	78	CT	Prediction of BRCA1-associated protein 1 mutation status in ccRCC	13.9 (5)
**Gill [27]**	2019	*Abdom Radiol (NY)*	83	CT	Differentiation of juxtatumoral perinephric fat surrounding high versus low grade (ISUP) ccRCC	0 (0)
**Gillingham [28]**	2019	*J Comput Assist Tomogr*	27	MRI	Discrimination of benign and malignant Bosniak IIF and III renal cysts	0 (0)
**Goh [29]**	2011	*Radiology*	39	CT	Treatment response to antiangiogenic therapy: Treatment-associated texture changes under TKI and correlation with time to progression	0 (0)
**Goyal [30]**	2019	*Abdom Radiol (NY)*	33	MRI	Differentiation of ccRCC versus non-ccRCC and high versus low grade RCC	13.9 (5)

**Haider [31]**	2017	*Cancer Imaging*	40	CT	Treatment response to antiangiogenic therapy: Treatment-associated texture changes under sunitinib and correlation with PFS and OS	8.3 (3)
**Han [32]**	2019	*Journal of Digital Imaging*	n.a.	CT	Discrimination of RCC subtypes (ccRCC, papRCC, chrRCC)	0 (-1)
**He [33]**	2020	*Acad Radiol*	227	CT	Machine learning based on ANN for prediction of tumor grade: High versus low grade ccRCC	16.7 (6)
**He [34]**	2019	*Medicine (Baltimore)*	227	CT	Prediction of tumor grade: High versus low grade ccRCC	13.9 (5)
**Hoang [35]**	2018	*Abdom Radiol (NY)*	41	MRI	Differentiation of benign and malignant renal masses (oncocytoma vs. ccRCC and papRCC) and between RCC subtypes (ccRCC vs. papRCC)	11.1 (4)
**Hodgon [36]**	2015	*Radiology*	100	CT	Differentiation of fpAML and RCC (multiple subtypes)	13.9 (5)
**Huhdanpaa [37]**	2015	*Abdom Imaging*	65	CT	Prediction of Fuhrman Grade: High versus low grade ccRCC	0 (-2)
**Khene [38]**	2018	*World J Urol*	70	CT	Prediction of adherent perinephric fat	12.5 (4.5)
**Kierans [39]**	2014	*AJR Am J Roentgenol*	61	MRI	Prediction of tumor grade: High versus low grade ccRCC	18.1 (6.5)
**Kim [40]**	2008	*Radiology*	144	CT	Differentiation of AMLwvf and RCC (multiple subtypes)	0 (-2)
**Kim [41]**	2019	*AJR Am J Roentgenol*	286	CT	Differentiation of low-attenuation RCC and benign renal cysts	11.1 (4)
**Kocak [42]**	2019	*Acta Radiol*	65	CT	Prediction of protein BAP1 mutation status in ccRCC	13.9 (5)
**Kocak [43]**	2019	*Eur Radiol*	47	CT	Influence of segmentation margin (contour-focused and margin shrinkage of 2mm) in ccRCC to predict tumor grade (nuclear grade as reference standard to compare reproducibility and classification performance of segmentation with contour-focus versus with margin shrinkage of 2mm)	13.9 (5)
**Kocak [44]**	2019	*AJR Am J Roentgenol*	81	CT	Prediction of tumor grade: High versus low grade ccRCC	20.8 (7.5)
**Kocak [45]**	2019	*AJR Am J Roentgenol*	45	CT	Prediction of PBRM1 mutation status in ccRCC	20.8 (7.5)

**Kocak [46]**	2019	*AJR Am J Roentgenol*	30	CT	Influence of Intra- and Interobserver Manual Segmentation Variability on Radiomic Feature Reproducibility (ccRCC)	30.6 (11)
**Kocak [47]**	2018	*Eur J Radiol*	94	CT	Differentiation of non-ccRCC versus ccRCC and ccRCC versus papRCC versus chrRCC	29.2 (10.5)
**Kunapuli [48]**	2018	*J Digit Imaging*	150	CT	Differentiation of benign and malignant renal lesions	15.3 (5.5)
**Lee [49]**	2018	*Med Phys*	80	CT	Differentiation of AMLwvf and ccRCC	8.3 (3)
**Lee [50]**	2017	*Med Phys*	50	CT	Differentiation of AMLwvf and ccRCC	15.3 (5.5)
**Lee [51]**	2020	*Cancers (Basel)*	58	CT	Prediction of post-operative metastases	38.9 (14)
**Leng [52]**	2017	*Abdom Radiol (NY)*	139	CT	Differentiation of ccRCC and papRCC and AML	12.5 (4.5)
**Li [53]**	2018	*AJR Am J Roentgenol*	92	MRI	Differentiation of ccRCC, papRCC, chrRCC, AMLwvf and oncocytoma	0 (0)
**Li [54]**	2019	*Acad Radiol*	140	MRI	Differentiation of AMLwvf and ccRCC	0 (0)
**Li [55]**	2019	*Abdom Radiol (NY)*	61	CT	Differentiation of chrRCC and oncocytoma	16.7 (6)
**Li [56]**	2019	*Eur Radiol*	255	CT	Differentiation of ccRCC versus non-ccRCC and association of imaging features with VHL mutation	41.7 (15)
**Lin [57]**	2019	*Abdom Radiol (NY)*	231	CT	Prediction of tumor grade: High versus low grade ccRCC	2.8 (1)
**Linguraru [58]**	2009	*Conf Proc IEEE Eng Med Biol Soc*	40	CT	Differentiation of benign versus malignant renal masses and other subtypes	16.7 (6)
**Linguraru [59]**	2011	*Med Phys*	43	CT	Differentiation of renal lesions associated with VHL, BHD, hereditary papRCC and hereditary leiomyomatosis and renal cancers	13.9 (5)
**Liu [60]**	2017	*Int J Clin Exp Med*	44	CT	Differentiation of renal primary undifferentiated pleomorphic sarcoma and RCC (ccRCC, papRCC, chrRCC)	13.9 (5)
**Lubner [61]**	2016	*AJR Am J Roentgenol*	157	CT	Association of texture features with histologic subtype (ccRCC, papRCC and chrRCC), nuclear grade, pathologic stage, and clinical outcome (time to disease recurrence and death due to disease)	12.5 (4.5)
**Ma [62]**	2020	*Abdom Radiol*	84	CT	Differentiation of fpAML and ccRCC	19.4 (7)

**Mains [63]**	2018	*Br J Radiol*	69	CT	Correlation with OS and PFS under various treatments	20.8 (7.5)
**Marigliano [64]**	2019	*Technol Cancer Res Treat*	20	CT	Correlation of texture analysis with the expression of selected oncogenic microRNAs	0 (0)
**Nazari [65]**	2020	*Radiol Med*	71	CT	Prediction of Fuhrman Grade: High versus low grade ccRCC	33.3 (12)
**Nie [66]**	2020	*Eur Radiol*	99	CT	Differentiation of AMLwvf and ccRCC	38.9 (14)
**Paschall [67]**	2018	*Abdom Radiol (NY)*	55	MRI	Differentiation of ccRCC versus papRCC and oncocytoma	0 (-1.5)
**Picard [68]**	2019	*J Comput Assist Tomogr*	n.a.	CT	Discrimination of renal cysts and RCC	0 (-1)
**Purkayastha [69]**	2020	*Sci Rep*	n.a.	MRI	Prediction of Fuhrman Grade: High versus low grade RCC	40.3 (14.5)
**Raman [70]**	2014	*Acad Radiol*	99	CT	Differentiation of ccRCC, papRCC, oncocytomas and renal cysts	19.4 (7)
**Ramesh [71]**	2018	*J Clin Diagn Res*	188	CT	Differentiation of normal renal tissue, benign and malignant renal masses	0 (-3)
**Reynolds [72]**	2018	*PLoS One*	12	MRI	Prediction of treatment response after SABR	15.3 (5.5)
**Said [73]**	2020	*Abdom Radiol (NY)*	125	MRI	Differentiation of benign renal lesions versus RCC and characterization of RCC subtypes (ccRCC and papRCC)	19.4 (7)
**Sasaguri [74]**	2015	*AJR Am J Roentgenol*	166	CT	Differentiation of oncocytoma versus RCC (papRCC and ccRCC and other subtypes)	19.4 (7)
**Schieda [75]**	2018	*AJR Am J Roentgenol*	37	CT	Prediction of tumor grade: High versus low grade chrRCC	15.3 (5.5)
**Schieda [76]**	2015	*AJR Am J Roentgenol*	35	CT	Differentiation of sarcomatoid RCC and ccRCC	15.3 (5.5)
**Scrima [77]**	2019	*Abdom Radiol (NY)*	249	CT	Differentiation of histologic subtype (ccRCC versus non-ccRCC), prediction of nuclear grade and correlation of texture analysis with DNA-expression levels	8.3 (3)
**Simpfendorfer [78]**	2009	*AJR Am J Roentgenol*	36	CT	Differentiation of AMLwvf and RCC	9.7 (3.5)
**Shu [79]**	2018	*Eur J Radiol*	260	CT	Prediction of Fuhrman Grade: High versus low grade ccRCC	29.2 (10.5)
**Shu [80]**	2019	*Eur J Radiol*	271	CT	Prediction of ISUP Grade: High versus low grade ccRCC	19.4 (7)
**Soma [81]**	2018	*Int J Urol*	126	CT and MRI	Differentiation of fpAML and RCC	0 (-2)

**Stanzione [82]**	2020	*J Digit Imaging*	32	MRI	Prediction of Fuhrman Grade: High versus low grade ccRCC	38.9 (14)
**Sun [83]**	2019	*Medicine (Baltimore)*	227	CT	Prediction of Fuhrman Grade: High versus low grade ccRCC	26.1 (13)
**Sun [84]**	2020	*AJR Am J Roentgenol*	290	CT	Differentiation of histologic RCC subtypes (ccRCC from papRCC and chrRCC) and differentiation of ccRCC from fpAML and oncocytoma and differentiation of papRCC and chrRCC from fpAML and oncocytomas	22.2 (8)
**Takahashi [85]**	2015	*AJR Am J Roentgenol*	153	CT	Differentiation of AMLwvf and RCC	13.9 (5)
**Takahashi [86]**	2016	*Abdom Radiol (NY)*	112	CT	Differentiation of AML and RCC	0 (-3)
**Tanaka [87]**	2011	*Int J Urol*	41	MRI	Differentiation of AMLwvf and RCC	0 (-2.5)
**Tanaka [88]**	2020	*AJR Am J Roentgenol*	159	CT	Differentiation of malignant and benign renal masses	11.1 (4)
**Tang [89]**	2020	*AJR Am J Roentgenol*	115	CT	Differentiation of fpAML and RCC	9.7 (3.5)
**Uhlig [90]**	2020	*Medicine (Baltimore)*	94	CT	Differentiation of malignant and benign renal masses	20.8 (7.5)
**Varghese [91]**	2018	*AJR Am J Roentgenol*	174	CT	Differentiation of malignant and benign renal masses (various subtypes)	16.7 (6)
**Varghese [92]**	2018	*Br J Radiol*	156	CT	Differentiation of malignant and benign renal masses (various subtypes)	0 (-0.5)
**Vendrami [93]**	2018	*AJR Am J Roentgenol*	41	MRI	Differentiation of papRCC type 1 and type 2	15.3 (5.5)
**Wang [94]**	2016	*Sci Rep*	21	MRI	Reproducibility of histogram parameters	18.1 (6.5)
**Wang [95]**	2020	*Eur Radiol*	77	MRI	Differentiation of RCC subtypes (ccRCC, papRCC and chrRCC)	0 (0)
**Xi [96]**	2018	*Eur Radiol*	16	MRI	Prediction of ISUP Grade: High versus low grade ccRCC	19.4 (7)
**Xi [97]**	2020	*Clin Cancer Res*	n.a.	MRI	Differentiation of malignant and benign renal masses	38.9 (14)
**Yan [98]**	2015	*Acad Radiology*	50	CT	Differentiation of ccRCC, papRCC and AML	13.9 (5)
**Yang [99]**	2019	*Mol Imaging*	56	CT	Differentiation of fpAML and chrRCC	19.4 (7)
**Yang [100]**	2020	*Eur Radiol*	163	CT	Differentiation of AMLvwf and RCC	18.1 (6.5)

**Yap [101]**	2018	*Urology*	150	CT	Differentiation of malignant and benign renal masses	0 (-1)
**Yasar [102]**	2020	*Abdom Radiol (NY)*	77	CT	Differentiation of histologic subtype (ccRCC versus non-ccRCC), Fuhrman grade and TNM stage	0 (-2)
**Yin [103]**	2018	*Clin Radiol*	8	PET/MRI	Differentiation of molecular subtypes ccA and ccB of ccRCC	22.2 (8)
**Yin [104]**	2017	*Sci Rep*	9	PET/MRI	Differentiation of expressions of angiogenesis and VEGF	11.1 (4)
**You [105]**	2019	*Clin Radiol*	67	CT	Differentiation of AMLvwf and ccRCC	8.3 (3)
**Yu [106]**	2017	*Abdom Radiol (NY)*	119	CT	Differentiation of ccRCC, papRCC, chrRCC and oncocytoma	0 (-0.5)
**Zabihollahy [107]**	2020	*Eur Radiol*	315	CT	Differentiation of benign and malignant RCC	16.7 (6)
**Zabihollahy [108]**	2020	*Med Phys*	315	CT	Automated localization of renal masses	36.1 (13)
**Zabihollahy [109]**	2020	*IEEE Access*	315	CT	Differentiation from benign renal cysts and solid renal masses	13.9 (5)
**Zhang [110]**	2019	*Clin Radiol*	127	CT	Differentiation of RCC subtypes (ccRCC versus non-ccRCC (papRCC and chrRCC))	19.4 (7)
**Zhang [111]**	2019	*J South Med Univ*	66	CT	Detection of renal tumors in patients with hydronephrosis and calculi	19.4 (7)
**Zhang [112]**	2015	*AJR Am J Roentgenol*	46	MRI	Prediction of Fuhrman Grade: High versus low grade ccRCC	0 (-1)
**Zhou [113]**	2019	*Trans Oncol*	192	CT	Differentiation of malignant and benign renal masses	38.9 (14)

Abbreviations: RQS = radiomics quality score, papRCC = papillary renal cell carcinoma, RCC = renal cell carcinoma, NSCLC = non-small cellular lung carcinoma, SCLC = small cell lung carcinoma, OS = overall survival, RECIST = Response evaluation criteria in solid tumors, VEGF = vascular endothelial growth factor, TKI = tyrosine kinase inhibitor, AMLwvf = angiomyolipoma without visible fat, ccRCC = clear-cell renal cell carcinoma, VHL = Von-Hippel-Lindau, PBRM1 = polybromo-1, BAP 1 = BRCA1 associated protein-1, AML = angiomyolipoma, chrRCC = chromophobe renal cell carcinoma, mpMRI = multiparametric magnetic resonance tomography, ISUP = International Society of Urologic Pathologists, PFS = progression free survival, ANN = artificial neural networks, pfAML = fat-poor angiomyolipoma, BHD = Birt-Hogg-Dubé, SABR = stereotactic ablative radiotherapy.

Supplementary Table S3: RQS [points] over time

Year	2008	2009	2010	2011	2012	2013	2014	2015	2016	2017	2018	2019	2020	All
**Number of studies [n (%)]**	2 (1.8)	2 (1.8)	0	3 (2.7)	2 (1.8)	1 (0.9)	3 (2.7)	10 (8.8)	5 (4.4)	8 (7.1)	24 (21.2)	30 (26.5)	23 (20.4)	113 (100)
**Mean**	-2.0	4.75	-	0.83	0.25	7	7.17	4.65	3.10	3.0	4.19	4.97	7.76	4.88
**Median**	-2.0	4.75	-	0.0	0.25	7	7.0	5.0	4.0	4.25	5.50	5.0	7.0	5.0
**SD**	0.0	1.77	-	3.82	0.35	n.a.	0.76	3.90	3.60	3.12	4.52	4.56	4.97	4.60
**Minimum**	-2.0	3.5	-	-2.5	0.0	7	6.5	-2.0	-3.0	-3.0	-3.0	-1.0	-2.0	-3.0
**Maximum**	-2.0	6.0	-	5.0	0.5	7	8.0	12.0	6.50	5.50	14	15.0	14.5	15.0
**IQR**	-2.0 – -2.0	3.5 – n.a.	-	-2.5 – n.a.	0.0 – n.a.	7 – 7	6.5 – n.a.	3.5 – 5.88	0.25 – 5.50	0.38 – 5.38	-0.38 – 7.0	0 – 7.13	5.0 – 13.0	0.25 – 7.0

Abbreviations: RQS = radiomics quality score, SD = standard deviation, IQR = interquartile range

Supplementary Table S4: Summary of studies investigating the dignity of renal masses (n = 52) and reasons for exclusion from the meta-analysis

Study	Year	Title	Number of patients	Modality	Gold standard	Research question and summary of methods	Main results and conclusions	Reasons for exclusion from the meta-analysis
**Differentiation of angiomyolipoma from RCC**								
**Catalano [7]**	**2008**	Pixel distribution analysis: can it be used to distinguish clear cell carcinomas from angiomyolipomas with minimal fat?	50	CT	Histology (not further specified if surgical or bioptic)	Differentiation of AMLwvf from ccRCC: Lesions were measured, and a histogram (number of pixels with each attenuation) was calculated electronically within a central ROI. The percentage of pixels below the attenuation thresholds -20 HU and 10 HU was calculated in both cohorts. The average percentage of subthreshold pixels at each threshold was compared.	No significant difference for all thresholds <0 HU could be found between both cohorts.	
**Chaudry [9]**	**2012**	Histogram analysis of small solid renal masses: differentiating minimal fat amgiomyolipoma from renal cell carcinoma.	64	CT	Histology (surgical or bioptic)	Differentiation of AMLwvf from ccRCC and papRCC: Using attenuation measurement histogram analysis, two blinded radiologists determined the percentage of negative pixels within each renal mass. The percentages of negative pixels below different attenuation thresholds were recorded. Radiologists and different cohorts were compared.	No significant difference in the percentage of negative pixels was found between AMLwvf versus ccRCC and AMLwvf and papRCC at any of the selected attenuation thresholds for either radiologist was found.	
**Cui [14]**	**2019**	Differentiation of renal angiomyolipoma without visible fat from renal cell carcinoma by machine learning based on whole-tumor computed tomography features.	171	CT	Histology (surgical)	Differentiation of AMLwvf from RCC: Texture features were extracted from whole-tumor images in three phases. A support vector machine with the recursive feature elimination method based on fivefold cross-validation with the synthetic minority oversampling technique (SMOTE) was utilized to establish classifiers for differentiation of cohorts. The performance of the classifiers based on three-phase and single-phase images were compared with each other and morphological interpretations.	A machine learning classifier achieved the best performance in differentiating AMLwvf from all RCC, ccRCC, and non-ccRCC. Morphological interpretations achieved lower performance.	Data augmentation using SMOTE (no true numbers given, only sensitivity and specificity for augmented cases).
**Deng (1) [16]**	**2020**	Usefulness of CT texture analysis in differentiating benign and malignant renal tumors.	501	CT	Histology (not further specified if surgical or bioptic)	Differentiation of RCC and benign renal tumors: A ROI was drawn encompassing the largest cross-section of the tumor on venous phase axial CT. Different texture analysis parameters were compared between cohorts.	Differences in entropy were helpful in differentiation RCC from AMLwvf, and chrRCC from oncocytoma.	
**Feng [22]**	**2018**	Machine learning-based quantitative texture analysis of CT images of small renal masses: Differentiation of	58	CT	Histology (surgical)	Differentiation of AMLwvf and RCC (multiple subtypes): Texture features were extracted from the largest possible tumorous ROIs by manual segmentation.	16 features showed significant intergroup differenced and had good interobserver agreement. An optimal feature subset including 11 features was further selected	

		angiomyolipoma without visible fat from renal cell carcinoma.”				The support vector machine with recursive feature elimination (SVM-RFE) and synthetic minority oversampling technique (SMOTE) were adopted to establish classifiers, and the performance of classifiers was assessed.	by the SVM-RFE method. The SVM-RFE+SMOTE classifier achieved the best performance in discriminating AMLwvf and RCC.	
**Gaing (1) [25]**	**2015**	Subtype differentiation of renal tumors using voxel-based histogram analysis of intravoxel incoherent motion parameters	44	MRI	Histology (surgical)	Differentiation of ccRCC, papRCC, chrRCC, oncocytoma and AML: Voxel-based histogram analysis of intravoxel incoherent motion imaging (IVIM) parameters. A biexponential model was fitted to the diffusion signal data using a segmented algorithm to extract different IVIM parameters for each voxel.	Two IVIM parameters differentiated 8 of 15 pairs of renal tumors. Histogram analysis of IVIM parameters differentiated 9 of 15 subtype pairs. Intravoxel incoherent motion imaging parameters with inclusion of histogram measures of heterogeneity can help differentiate malignant from benign lesions as well as various subtypes of RCC.	Insufficient disclosure of the results: No AUC, sensitivity or specificity given, only comparison of histogram distribution parameters (perfusion fraction, tissue diffusivity, pseudodiffusivity).
**Hodgdon [36]**	**2015**	Can Quantitative CT Texture Analysis be Used to Differentiate Fat-poor Renal Angiomyolipoma from Renal Cell Carcinoma on Unenhanced CT Images?	100	CT	Histology (not further specified if surgical or bioptic)	Differentiation of AMLwvf and RCC (multiple subtypes): Axial unenhanced CT images were manually segmented. Texture features related to the gray-level histogram, gray-level occurrence, and run-length matrix statistics were evaluated. The most discriminative features were used to generate support vector machine (SVM) classifiers. Heterogeneity of the lesions was subjectively graded on a five-point scale.	There was lower lesion homogeneity and higher lesions entropy in RCCs. A model incorporating several texture features resulted in a high performance. Each of the three textural-based classifiers was more accurate than radiologists’ subjective heterogeneity ratings.	
**Kim [40]**	**2008**	CT histogram analysis: differentiation of angiomyolipoma without visible fat from renal cell carcinoma at CT imaging.	144	CT	Histology (not further specified if surgical or bioptic)	Differentiation of AMLwvf and RCC (multiple subtypes): The percentage of voxels and pixels of unenhanced CT histograms were compared in the two groups according to the CT number categories.	The percentages of voxels and pixels with a CT number less than -30 HU, less than -20 HU, less than -10 HU, and less than 0 HU were greater in the AMLwvf group. CT histogram analysis may be useful for differentiating AMLwvf from RCC.	
**Lee [49]**	**2018**	Deep feature classification of angiomyolipoma without visible fat and renal cell carcinoma in abdominal contrast-enhanced CT images with texture image patches and hand-crafted feature concatenation.	80	CT	Histology (not further specified if surgical or bioptic)	Differentiation of AMLwvf and ccRCC and development of an automatic deep feature classification method for distinguishing AMLwvf from RCC: First, hand-crafted features were extracted from the tumor contours. Second, deep features were extracted from the ImageNet pretrained deep learning model with the SRM image patches. In deep feature extraction, texture image patches (TIP) were proposed. Finally, the two featured were concatenated and the random forest classifier was trained on these concatenated features to classify the tumor subtypes.	The proposed shape features and TIPs improved the hand-crafted features and deep features, and the feature concatenation further enhanced the quality of features for differentiating AMLwvf from ccRCC.	Multiple publications deriving from one study. Only the one with better methodological quality according to the RQS was included (Lee 2017).
**Lee [50]**	**2017**	Differentiation of fat-poor angiomyolipoma from clear	50	CT	Histology (not further	Differentiation of AMLwvf and ccRCC and development of a computer-aided	From three selection methods, three histogram features were jointly selected as	

		cell renal cell carcinoma in contrast-enhanced MDCT images using quantitative feature classification.			specified if surgical or bioptic)	classification system to differentiate AMLwvf from ccRCC: Tumors were manually segmented, quantitative image features were extracted, a number of feature selection methods were applied, and finally, the feature classifiers were trained.	key features to distinguish two types of renal masses. In feature classification, two classifiers demonstrated with one type of feature selection demonstrated the best performance.	
**Leng [52]**	**2017**	Subjective and objective heterogeneity scores for differentitating small renal masses using contrast enhanced CT	139	CT	Histology (surgical)	Differentiation of ccRCC and papRCC and AML: A representative contrast-enhanced CT image for each mass was selected, a largest possible ROI was manually drawn from which three objective heterogeneity indices were calculated. Objective heterogeneity indices were also calculated after images were processed with a denoising algorithm. Two radiologists also subjectively scored each mass according to their subjective heterogeneity.	Both subjective and objective heterogeneity indices can differentiate ccRCC from papRCC and AML. Noise reduction improved differentiation of ccRCC from papRCC, but not differentiation of AML from ccRCC.	Insufficient disclosure of the results: AUC given, but no sensitivity or specificity.
**Li (1) [114]**	**2018**	Subtype Differentiation of Small (<= 4 cm) Solid Renal Mass Using Volumetric Histogram Analysis of DWI at 3-T MRI.	92	MRI	Histology (surgical)	Differentiation of ccRCC, papRCC, chrRCC, AMLwvf and oncocytoma: Volumetric ADC maps were generated using all sliced of reduced-FOV DW images to obtain different histogram parameters.	ADC histogram parameters differentiated eight of 10 pairs of renal tumors.	Insufficient disclosure of the results: No AUC, sensitivity or specificity given, only comparison of histogram distribution parameters (ADC).
**Li [54]**	**2019**	Whole-Tumor Quantitative Apparent Diffusion Coefficient Histogram and Texture Analysis to Differentiation of Minimal Fat Angiomyolipma from Clear Cell Renal Cell Carcinoma.	140	MRI	Histology (not further specified if surgical or bioptic)	Differentiation of AMLwvf and ccRCC: Whole-tumor ROIs were drawn on all sliced of diffusion-weighted imaging to obtain histogram and texture parameters, which were compared between groups.	Some of the parameters differed significantly between groups (lower mean ADC, median ADC, 10th, 25^{th,} 75th, 90th percentiles ADC, and skewness).	
**Ma [62]**	**2020**	Can whole-tumor radiomics-based CT analysis better differentiate fat-poor angiomyolipoma from clear cell renal cell carcinoma compared with conventional CT analysis?	84	CT	Histology (surgical)	Differentiation of AMLwvf and ccRCC: Whole-tumor ROIs were contoured, radiomic features were dimensionally reduced, and four radiomics logistic classifiers were built. After collecting the qualitative and quantitative conventional CT characteristics, the conventional CT analysis logistic classifier and radiomics-based logistic classifier were constructed.	Whole-tumor radiomics-based CT analysis was superior to conventional CT analysis. Cyst degeneration, pseudo capsule, and sum rad-score were the most significant factors.	Insufficient disclosure of the results: AUC given, but no sensitivity or specificity.
**Nie [66]**	**2020**	A CT-based radiomics normogram for differentiation of renal angiomyolipoma without visible fat from	99	CT	Histology (surgical)	Differentiation of AMLwvf and ccRCC: The cohort was divided into a training and a validation set. A radiomics signature was constructed and a radiomics score (Rad-score) was calculated. Demographics and CT findings were assessed to build a	The CT-based radiomics normogram demonstrated favorable predictive efficacy for differentiating AMLwvf from ccRCC.	

		homogeneous clear cell renal cell carcinoma.				clinical factors model. Combined with the Rad-score and independent clinical factors, a radiomics normogram was constructed.		
**Simpfendorfer [78]**	**2009**	Attenuation Pixels Aid Diagnosis?	36	CT	Histology (surgical)	Differentiation of AMLwvf and RCC: Three radiologists counted the number of pixels with attenuation less than -10, -20, and -30 HU. Analysis of the number of pixels at each cutoff was performed.	CT findings of more than 20 pixels with attenuation less than -20 HU and more than 5 pixels with attenuation less than -30 HU have a positive predictive value of 100% in detection AML, but most AMLwvf cannot be reliably identified on the basis of an absolute pixel count.	
**Soma [81]**	**2018**	Potential for computer-aided diagnosis using a convolutional neural network algorithm to diagnose fat-poor angiomyolipoma in enhanced computed tomography and T2-weighted magnetic resonance imaging	126	CT and MRI	Histology (surgical or bioptic)	Differentiation of AMLwvf and RCC via the use of computer-aided diagnosis (CAD) with a convolutional neural network (CNN) algorithm: The contrast information was converted to information using a histogram smoothing algorithm. Segmentation was manually conducted, resized, and normalized. Patients were divided into test and training dataset without data augmentation. LeNet was used, a seven-level convolutional network for reproducible simple structure for prototyping.	The CAD system that uses CNNs for AMLwvf showed potential to provide reproducible interpretation, and a greater level of standardization and consistency.	Insufficient disclosure of the results: No numbers given.
**Takahashi [85]**	**2015**	Small (< 4 cm) Renal Masses: Differentiation of Angiomyolipoma Without Visible Fat From Renal Cell Carcinoma Using Unenhanced and Contrast-Enhanced CT.	153	CT	Histology (surgical)	Differentiation of AMLwvf and RCC: Assessment of demographic data and size, shape, CT attenuation, and heterogeneity of the renal mass on unenhanced and contrast-enhanced CT. Development of different models including different demographic and various CT findings for contrast-enhanced CT and unenhanced CT.	Combination of various CT and demographic findings allowed differentiation of AML from RCC. Sensitivity for differentiation of AML from RCC was better for the combined unenhanced and contrast-enhanced CT-based model compared to the contrast-enhanced based model.	
**Takahashi [86]**	**2016**	CT negative attenuation pixel distribution and texture analysis for detection of fat in small angiomyolipoma on unenhanced CT.	112	CT	Histology (surgical)	Differentiation of AML and RCC: A ROI was manually placed over a renal mass on unenhanced CT. In-house software generated multiple overlapping small-ROIs of various sized within whole-lesion ROI. Maximal number of pixels under cut-off attenuation values in the multiple small-ROIs was calculated. Skewness of CT attenuation histogram was calculated from whole-lesion-ROI. Presence of fat was evaluated subjectively. Performance of subjective and objective methods for identifying fat was compared.	CT negative attenuation pixel distribution did not identify fat in AML beyond subjective evaluation. Addition of skewness by texture analysis improved identifying fat in AML.	Multiple publications deriving from one study. Only the one with better methodological quality according to the RQS was included (Takahashi 2015).
**Tanaka [87]**	**2011**	Diffusion-weighted magnetic resonance imaging	41	MRI	Histology (surgical)	Differentiation of AMLwvf and RCC: The signals of the tumors on DW-MRI were	Most of the ccRCC exhibited a heterogenous signal on DW-MRI and	Insufficient disclosure of the results: No AUC, sensitivity

		in the differentiation of angiomylipoma with minimal fat from clear cell renal cell carcinoma.				analyzed subjectively and the ADC values and histograms were assessed objectively.	several peaks in the ADC value histogram, whereas most of the AMLwvf exhibited a homogeneous signal on DW-MRI and a single prominent peak in the histogram. The standard deviations of the ADC values were smaller for AMLwvf than for ccRCC.	or specificity given, only comparison of histogram distribution parameters (ADC).
**Tang [89]**	**2020**	Quantitative Analysis of Multiphase Contrast-Enhanced CT Images: A Pilot Study of Preoperative Prediction of Fat-Poor Angiomyolipoma and Renal Cell Carcinoma.	115	CT	Histology (surgical)	Differentiation of AMLwvf and RCC: Division of the cohort into train and test set after data augmentation. High-dimensional histogram-based features, texture-based features, and Laws features were extracted from CT and combined as different combination sets to construct a prediction model based on the least absolute shrinkage and selection operator procedure for the prediction of AMLwvf and RCC. In addition, the effects of different gray-scales of quantitative features on prediction performance was investigated.	Histogram-based features, histogram-based features and texture-based features, histogram-based features and Laws features, and histogram-based features, texture-based features, and Laws features achieved satisfying performances in the test set. The different quantitative gray-scales did not have an obvious effect on prediction performances.	Data augmentation: each ROI split into two samples to create training and validation set.
**Varghese (1) [91]**	**2018**	Differentiation of Predominantly Solid Enhancing Lipid-Poor Renal Cell Masses by Use of Contrast-Enhanced CT: Evaluating the Role of Texture in Tumor Subtyping.	174	CT	Histology (surgical)	Differentiation of malignant and benign renal masses (various subtypes): Whole lesions were manually segmented and coregistered from the multiphase contrast-enhanced CT (CECT) scans. CECT images of the renal masses were used as inputs to a CECT texture analysis panel comprising 31 texture metrics derived with six texture methods. Stepwise logistic regression analysis was used to select the best predictor from each of the texture methods and performance was assessed.	Entropy, entropy of fast-Fourier transform magnitude, mean, uniformity, information measure of correlation 2, and sum of averages were among the texture predictors aiding renal mass subtyping. The overall CECT-based tumor texture model was accurate for differentiating benign from malignant solid enhancing lipid-poor renal masses.	Insufficient disclosure of the results: AUC given, but no sensitivity or specificity.
**Varghese (1) [92]**	**2018**	Differentiating solid, non-macroscopic fat containing, enhancing renal masses using Fourier analysis of multiphase CT.	156	CT	Histology (surgical)	Differentiation of malignant and benign renal masses (various subtypes): Whole lesions were manually segmented using Synapse 3D (Fujifilm, CT) and co-registered from the multiphase CT acquisitions for each tumor to test the feasibility of two-dimensional fast Fourier transforms (FFT)-based imaging metrics for differentiating solid, non-macroscopic fat containing, enhancing renal masses in contrast-enhanced CT images. Matlab function, FFT2 was used to perform the image to frequency transformation.	FFT-based metrics were different between 1. benign versus malignant renal masses, 2. oncocytoma versus ccRCC, and 3. oncocytoma versus AMLwvf.	Insufficient disclosure of the results: AUC given, but no sensitivity or specificity.

**Yan [98]**	**2015**	Angiomyolipoma with minimal fat: differentiation from clear cell renal cell carcinoma and papillary renal cell carcinoma by texture analysis on CT images.	50	CT	Histology (surgical or bioptic)	Differentiation of ccRCC, papRCC and AML: Unenhanced and contrast-enhanced CT images were analyzed and classified with a texture analysis software (MaZda). Tumor attenuation values and enhancement degree was determined by a ROI. Texture classification was performed for AMLwvf versus ccRCC, AMLwvf versus papRCC, and ccRCC versus papRCC.	Texture analysis enabled a reliable method for the discrimination of all three groups (AMLwvf, ccRCC, and papRCC). A trend toward better classification was observed with precontrast phase CT for the discrimination for AMLwvf versus ccRCC.	Insufficient disclosure of the results: No AUC, sensitivity or specificity given, only comparison of histogram distribution parameters (ADC).
**Yang [99]**	**2019**	Contrast-Enhanced CT Texture Analysis for Distinguishing Fat-Poor Renal Angiomyolipoma From Chromophobe Renal Cell Carcinoma.	56	CT	Histology (not further specified if surgical or bioptic)	Differentiation of AMLwvf and chrRCC: Texture features were extracted from 2D and 3D regions in triphasic CT images. The 2D and 3D texture analysis models were constructed with the least absolute shrinkage and selection operator algorithm and texture scores were calculated. The diagnostic performance of the models was evaluated with respect to calibration, discrimination, and clinical usefulness.	Five features 2D features and eight 3D features were selected to build the respective models. Both models showed good discrimination and calibration. The 3D model was better compared to the 2D model regarding clinical usefulness.	
**Yang [100]**	**2020**	Radiomics of small renal masses on multiphasic CT: accuracy of machine learning-based classification models for the differentiation of renal cell carcinoma and angiomyolipoma without visible fat.	163	CT	Histology (not further specified if surgical or bioptic)	Differentiation of AMLvwf and RCC: Manual segmentation of the ROI and feature extraction was performed on a representative slice with the largest lesion area on each phase of four-phase CT images. Features were fed into multiple classification models (built with classifiers and feature selection methods) and classification performances of the discriminative models were compared.	Image features extracted from the unenhanced phase CT image demonstrated dominant classification performances over features from other three phases. Two discriminative models (SVM + t_score and SVW + relief) achieved the highest classification performance.	
**You [105]**	**2019**	The value of quantitative CT texture analysis in differentiation of angiomylipoma without visible fat from clear cell renal cell carcinoma on four-phase contrast-enhanced CT images."	67	CT	Histology (not further specified if surgical or bioptic)	Differentiation of AMLvwf and ccRCC: The histogram, grey-level co-occurrence matrix, and grey-level run length matrix were evaluated on four-phase CT images. Sequential feature selection (SFS) and support vector machine (SVM) classifier with leave-one-out cross validation were used.	Using the SFS and SVM classifiers, five texture features were selected. Diagnostic performance for discrimination of AMLwvf from ccRCC of the five selected texture features for both unenhanced and contrast-enhanced CT phases was high.	
**Differentiation of oncocytoma from RCC**								
**Chen [10]**	**2017**	Voxel-based whole-lesion enhancement parameters: a study of its clinical value in differentiating clear cell renal cell carcinoma from renal oncocytoma	94	CT	Histology (surgical)	Differentiation of ccRCC from renal oncocytoma: Multiphase CT images were transferred to a three-dimensional workstation and whole lesion ROIs were manually segmented. Whole lesion enhancement and histogram distribution parameters skewness, kurtosis, standard deviation, and interquartile range were calculated. Whole lesion enhancement	Whole lesion enhancement alone did not demonstrate an advantage in discriminating between ccRCC and oncocytoma, but when combined with histogram distribution parameters, it did demonstrate a slight improvement.	Insufficient disclosure of the results: AUC given, but no sensitivity or specificity.

						parameters were compared to single ROI-based enhancement.		
**Coy [13]**	**2019**	Deep learning and radiomics: the utility of Google TensorFlow^{TM} Inception in classifying clear cell renal cell carcinoma and oncocytoma on multiphasic CT.	179	CT	Histology (not further specified if surgical or bioptic)	Differentiation of ccRCC and oncocytoma: The renal mass was contoured in each of four CT phases, resulting in a 3D volume of interest (VOI). Different approaches to convert the acquired VOI data into a set of images that adequately represented each tumor were investigated and used to train the final layer of the neural network model.	The best classification results was obtained in the excretory phase among thirteen classification methods tested.	
**Deng (2) [16]**	**2020**	Usefulness of CT texture analysis in differentiating benign and malignant renal tumors.	501	CT	Histology (not further specified if surgical or bioptic)	Differentiation of RCC and benign renal tumors: A ROI was drawn encompassing the largest cross-section of the tumor on venous phase axial CT. Different texture analysis parameters were compared between cohorts.	Differences in entropy were helpful in differentiation RCC from AMLwvf, and chrRCC from oncocytoma.	
**Gaing (2) [25]**	**2015**	Subtype differentiation of renal tumors using voxel-based histogram analysis of intravoxel incoherent motion parameters	44	MRI	Histology (surgical)	Differentiation of ccRCC, papRCC, chrRCC, oncocytoma and AML: Voxel-based histogram analysis of intravoxel incoherent motion imaging (IVIM) parameters. A biexponential model was fitted to the diffusion signal data using a segmented algorithm to extract different IVIM parameters for each voxel.	Two IVIM parameters differentiated 8 of 15 pairs of renal tumors. Histogram analysis of IVIM parameters differentiated 9 of 15 subtype pairs. Intravoxel incoherent motion imaging parameters with inclusion of histogram measures of heterogeneity can help differentiate malignant from benign lesions as well as various subtypes of RCC.	Insufficient disclosure of the results: No AUC, sensitivity or specificity given, only comparison of histogram distribution parameters (perfusion fraction, tissue diffusivity, pseudodiffusivity).
**Hoang [35]**	**2018**	Assessment of multiphasic contrast-enhanced MR textures in differentiating small renal mass subtypes	41	MRI	Histology (surgical)	Differentiation of benign and malignant renal masses (oncocytoma vs. ccRCC and papRCC) and between RCC subtypes (ccRCC vs. papRCC): Texture features were extracted from entire cross-sectional tumoral region in three consecutive slices containing the largest cross-sectional area from each of the four phases. The change in imaging feature between precontrast imaging and each postcontrast phase was calculated. Data dimension reduction and feature selection were performed by conducting followed by modified false discovery rate adjustment, and Lasso regression. Mutlivariate modeling incorporating the selected features was performed.	Histogram imaging features were informative variables in differentiating between benign and malignant masses, while texture imaging features were of added value in differentiating between subtypes of RCCs.	
**Li (2) [114]**	**2018**	Subtype Differentiation of Small (<= 4 cm) Solid Renal Mass Using Volumetric	92	MRI	Histology (surgical)	Differentiation of ccRCC, papRCC, chrRCC, AMLwvf and oncocytoma: Volumentric ADC maps were generated using all sliced of reduced-FOV DW	ADC histogram parameters differentiated eight of 10 pairs of renal tumors.	Insufficient disclosure of the results: No AUC, sensitivity or specificity given, only comparison of histogram

		Histogram Analysis of DWI at 3-T MRI.				images to obtain different histogram parameters.		distribution parameters (ADC).
**Li [55]**	**2019**	Value of radiomics in differential diagnosis of chromophobe renal cell carcinoma and renal oncocytoma.	61	CT	Histology (surgical)	Differentiation of chrRCC and oncocytoma: Volumes of interest (VOIs), including lesions on the images, were manually delineated using the RadCloud platform. A LASSO regression algorithm was used to screen the image features extracted from all VOIs. Five machine learning classifications were trained to distinguish chrRCC from oncocytoma by using a fivefold cross-validation strategy. The performance of the classifier was evaluated.	1029 features were extracted from all CT phases in total. The LASSO regression algorithm was used to screen out the best features for the different CT phases and for combined CT phases. All five classifiers had good diagnostic performance, the support vector machine classifier showed the best performance.	
**Paschall [67]**	**2018**	Differentiating papillary type I RCC from clear cell RCC and oncocytoma: application of whole-lesion volumetric ADC measurement.	55	MRI	Histology (not further specified if surgical or bioptic)	Differentiation of ccRCC versus papRCC and oncocytoma: Whole lesion measurements were performed and mean, median, skewness, kurtosis, and every 5th percentile ADCs were determined from the whole lesion histogram. Linear mixed models that accounted for within-subject correlation of lesion were used to ADCs among RCC subtypes. ROC curve analysis with optimal cutoff points was used to test the ability to the different groups.	Whole-lesion ADC values were significantly different between papRCC and ccRCC, and between papRCC and oncocytoma, demonstrating strong ability to differentiate subtypes across the quantiles. Best percentile RAC analysis demonstrated best AUC values for ccRCC versus papRCC and oncocytoma versus papRCC.	
**Raman (1) [70]**	**2014**	CT texture analysis of renal masses: pilot study using radom forest classification for prediction of pathology	99	CT	Histology (surgical)	Differentiation of ccRCC, papRCC, oncocytomas and renal cysts: ROIs were drawn around each mass on multiple slices in different phases of contrast-enhanced CT images. Unfiltered images and spatial band-pass filtered images were analyzed to quantify heterogeneity. A predictive model using quantitative parameters was constructed and externally validated.	Various renal masses (oncocytomas, ccRCC, cysts, and papRCC) were accurately classified using quantitative information derived from routine scans.	
**Sasaguri [74]**	**2015**	Small (< 4 cm) Renal Mass Differentiation of Oncocytoma From Renal Cell Carcinoma on Biphasic Contrast-Enhanced CT	166	CT	Histology (surgical)	Differentiation of oncocytoma versus RCC (papRCC and ccRCC and other subtypes): Patient demographics and CT tumor characteristics were evaluated. A multinomial logistic regression model was then constructed for differentiating oncocytoma from ccRCC and other subtype RCCs from papRCC, The probability of each group was calculated from the model. Diagnostic performance among three pairwise diagnoses and between oncocytoma and any RCC were assessed by AUC values.	Patient age, tumor CT attenuation values and skewness in both the corticomedullary and nephrogenic phases, and subjective tumor heterogeneity were significant variables in the multinomial logistic regression analysis. The logistic regression model using the variables showed the best AUC for the discrimination of oncocytomas from papRCCs.	

**Varghese (2) [91]**	**2018**	Differentiation of Predominantly Solid Enhancing Lipid-Poor Renal Cell Masses by Use of Contrast-Enhanced CT: Evaluating the Role of Texture in Tumor Subtyping.	174	CT	Histology (surgical)	Differentiation of malignant and benign renal masses (various subtypes): Whole lesions were manually segmented and coregistered from the multiphase contrast-enhanced CT (CECT) scans. CECT images of the renal masses were used as inputs to a CECT texture analysis panel comprising 31 texture metrics derived with six texture methods. Stepwise logistic regression analysis was used to select the best predictor from each of the texture methods and performance was assessed.	Entropy, entropy of fast-Fourier transform magnitude, mean, uniformity, information measure of correlation 2, and sum of averages were among the texture predictors aiding renal mass subtyping. The overall CECT-based tumor texture model was accurate for differentiating benign from malignant solid enhancing lipid-poor renal masses.	Insufficient disclosure of the results: AUC given, but no sensitivity or specificity.
**Varghese (2) [92]**	**2018**	Differentiating solid, non-macroscopic fat containing, enhancing renal masses using Fourier analysis of multiphase CT.	156	CT	Histology (surgical)	Differentiation of malignant and benign renal masses (various subtypes): Whole lesions were manually segmented using Synapse 3D (Fujifilm, CT) and co-registered from the multiphase CT acquisitions for each tumor to test the feasibility of two-dimensional fast Fourier transforms (FFT)-based imaging metrics for differentiating solid, non-macroscopic fat containing, enhancing renal masses in contrast-enhanced CT images. Matlab function, FFT2 was used to perform the image to frequency transformation.	FFT-based metrics were different between 1. benign versus malignant renal masses, 2. oncocytoma versus ccRCC, and 3. oncocytoma versus AMLwvf.	Insufficient disclosure of the results: AUC given, but no sensitivity or specificity.
**Yu [106]**	**2017**	Texture analysis as a radiomic marker for differentiating renal tumors.	119	CT	Histology (surgical)	Differentiation of ccRCC, papRCC, chrRCC and oncocytoma: Images were manually segmented, and texture analysis of the segmented tumors was performed. A support vector machine (SVM) method was also applied to classify tumor types. Texture analysis results were compared to the various tumors and AUCs were calculated. Similar calculations were performed with the SVM data.	Excellent discriminators of tumors were identified among the histogram-based features noting features skewness and kurtosis for differentiating ccRCC from oncocytoma. Histogram feature median demonstrated a high AUC for differentiating papRCCC from oncocytoma and other tumors. Machine learning further improved the results achieving very good to excellent discrimination of tumor subtypes. The ability of machine learning to distinguish ccRCC from other tumors was excellent.	Insufficient disclosure of the results: AUC given, but no sensitivity or specificity.

**Differentiation of not further specified benign renal tumors from RCC**								
**Deng (3) [16]**	**2020**	Usefulness of CT texture analysis in differentiating benign and malignant renal tumors.	501	CT	Histology (not further specified if surgical or bioptic)	Differentiation of RCC and benign renal tumors: A ROI was drawn encompassing the largest cross-section of the tumor on venous phase axial CT. Different texture analysis parameters were compared between cohorts.	Differences in entropy were helpful in differentiation RCC from AMLwvf, and chrRCC from oncocytoma.	
**Erdim [21]**	**2020**	Prediction of Benign and Malignant Solid Renal Masses: Machine Learning-Based CT Texture Analysis.	79	CT	Histology (surgical)	Differentiation of benign and malignant renal tumors (various subtypes) by 8 machine learning algorithms after manual segmentation of the ROI. Feature selection was performed using a nested-approch, results were compared to the respective area under the curve (AUC).	CT texture analysis via machine learning algorithms using a variety of features can differentiate RCC from benign masses with good reproducibility	
**Gillingham [28]**	**2019**	Bosniak IIF and III Renal Cysts: Can Apparent Diffusion Coefficient-Derived Texture Features Discriminate Between Malignant and Benign IIF and III Cysts?	27	MRI	Histology (not further specified if surgical or bioptic)	Discrimination of benign and malignant Bosniak IIF and III renal cysts using diffusion coefficient maps on MRI scans. After manual segmentation renal cystic lesions were evaluated by 8 different diffusion derived features.	MRI-diffusion coefficient-derived texture measures aid in predicting malignancy in Bosniak IIF and III cystic lesions.	Insufficient disclosure of the results: No AUC, sensitivity or specificity given, only comparison of histogram distribution parameters.
**Kim [41]**	**2019**	Utility of CT Texture Analysis in Differentiating Low-Attenuation Renal Cell Carcinoma From Cysts: A Bi-Insitutional Retrospective Study	286	CT	Histology (not further specified if surgical or bioptic) + fulfillment of predefined, radiographic criteria for benign cysts	Differentiation of low-attenuation RCC and benign renal cysts using CT texture analysis. ROI was automatically defined by a commercially available texture analysis program after preselecting a single section depicting the lesions' largest diameter. Results were compared to 2 novice and 2 expert readers.	Best results in differentiating RCC from cysts were derived by a combined model including mean gray-level attenuation, coarse entropy and kurtosis.	
**Kunapuli [48]**	**2018**	A Decision-Support Tool for Renal Mass classification.	150	CT	Histology (surgical)	Differentiation of benign and malignant renal lesions applying relational gradient boosting compared to standard machine-learning algorithms. Extensive feature reduction was performed to include 10 out of 204 radiomics features in the analysis.	Specialized relational machine learning algorithms succeeds in accurately predict malignancy in renal masses.	Insufficient disclosure of the results: AUC given, but no sensitivity or specificity.
**Li (3) [114]**	**2018**	Subtype Differentiation of Small (<= 4 cm) Solid Renal Mass Using Volumetric Histogram Analysis of DWI at 3-T MRI.	92	MRI	Histology (surgical)	Differentiation of ccRCC, papRCC, chrRCC, AMLwvf and oncocytoma: Volumentric ADC maps were generated using all sliced of reduced-FOV DW images to obtain different histogram parameters.	ADC histogram parameters differentiated eight of 10 pairs of renal tumors.	
**Linguraru [58]**	**2009**	Computer-aided renal cancer quantification and classification from contrast enhanced CT via histograms of curvature-related features."	40	CT	Histology (not further specified if surgical or bioptic)	Differentiation of benign versus malignant renal masses and other subtypes using curvature-related features after computer-assisted segmentation in portal venous phase. Enhancement in different phases was used to discriminate between tumor subtypes.	Computer-assisted evaluation of renal lesions is a promising tool for diagnosis of renal tumor subtype differentiation.	
**Picard [68]**	**2019**	Combined Qualitative and Quantitative Assessment of Low-Attenuation Renal Lesions Improves	n.a.	CT	Histology (surgical)	Discrimination of renal cysts and RCC using non-contrast CT images. A comparison of qualitative (3 readers, differing in level of experience) and quantitative (commercially	Combination of qualitative and quantitative analysis of non-contrast CT scans showed best performance in diagnosis of RCC.	

		Identification of Renal Malignancy on Noncontrast Computed Tomography.”				available texture analysis software) assessment was conducted.		
**Raman (2) [70]**	**2014**	CT texture analysis of renal masses: pilot study using radom forest classification for prediction of pathology	99	CT	Histology (surgical)	Differentiation of ccRCC, papRCC, oncocytomas and renal cysts: ROIs were drawn around each mass on multiple slices in different phases of contrast-enhanced CT images. Unfiltered images and spatial band-pass filtered images were analyzed to quantify heterogeneity. A predictive model using quantitative parameters was constructed and externally validated.	Various renal masses (oncocytomas, ccRCC, cysts, and papRCC) were accurately classified using quantitative information derived from routine scans.	
**Ramesh [71]**	**2018**	Assessment of primary solid renal mass using texture analysis of CT images of kidney by active contour method: A novel method.”	188	CT	Histology (surgical or bioptic)	Differentiation of normal renal tissue, benign and malignant renal masses from CT scans. Segmentation was performed using active contour method, data extraction based on co-occurrence matrices was analyzed by MATLAB software. Results were compared between intervention and control group.	Using radiomics features entropy, energy, sum average, sum variance, inertia and low gray level emphasis is a promising tool in differentiating the texture composition of renal masses.	Insufficient disclosure of the results: No AUC, sensitivity or specificity given, only comparison of histogram distribution parameters.
**Said [73]**	**2020**	Characterization of solid renal neoplasms using MRI-based quantitative radiomics features.	125	MRI	Histology (surgical)	Differentiation of benign renal lesions versus RCC and characterization of RCC subtypes (ccRCC and papRCC) via machine-learning algorithms on MRI-based radiomics features. Qualitative assessment was conducted by 2 radiologists and results compared to quantitative analysis. Radiomics software MATLAB was developed extracting 50 histogram and 140 texture features per lesion.	Diagnosis of renal masses can be aided by machine-learning algorithms using texture analysis on MRI-scans.	
**Sun [84]**	**2020**	Radiologic-Radiomic Machine Learning Models for Differentiation of Benign and Malignant Solid Renal Masses: Comparison With Expert-Level Radiologists.	227	CT	Histology (surgical)	Differentiating malignant from benign renal masses using machine learning algorithms in comparison to experienced radiologist raters. Semi-automated segmentation and manual identification of the ROI was conducted to design a 3D volume of interest. A variety of first-order intensity, shape and gray-level features were extracted and a support vector machine was applied to train for machine learning.e4for machine learning.e4	Qualitative assessment by expert radiologists showed a high interrater variability in discriminating malignant and benign renal masses, while machine learning may contribute to diagnostic accuracy.	
**Tanaka [88]**	**2020**	Differentiation of Small (<= 4 cm) Renal Masses on Multiphase Contrast-Enhanced CT by Deep Learning.	159	CT	Histology (surgical or bioptic)	Differentiation of malignant and benign renal masses (not further specified) was analyzed using convolutional neural network (CNN) model as deep learning tool. The study population was divided into training and validation groups and data augmentation was applied. Logistic regression models determined the predictive value of CNN models compared to patient data.	Coricomedullary phase image data allowed best prediction of malignancy in small renal masses using deep learning models.	Insufficient disclosure of the results: Data was divided randomly into 5 subsets (4 for augmentation and training, 1 for testing), total of 136 malign and 32 benign lesions, no numbers given for test dataset.

**Uhlig [90]**	**2020**	Discrimination malignant and benign clinical T1 masses on computed tomography: A pragmatic radiomics and machine learning approach	94	CT	Histology (surgical)	The study aimed at differentiation of malignant and benign T1 renal masses by CT-texture analysis with the help of machine learning algorithms. Segmentation was performed manually and a total of 120 radiomics features was derived from the image data. Results were compared to qualitative assessment by 2 radiologists. Receiver-operating characteristic curves and respective area under the curve present the extent of accuracy.	Compared to qualitative assessment radiomics combined with machine learning algorithms enables higher diagnostic accuracy in discriminating between malignant and benign renal lesions.	
**Varghese (3) [91]**	**2018**	Differentiation of Predominantly Solid Enhancing Lipid-Poor Renal Cell Masses by Use of Contrast-Enhanced CT: Evaluating the Role of Texture in Tumor Subtyping.	174	CT	Histology (surgical)	Differentiation of malignant and benign renal masses (various subtypes): Whole lesions were manually segmented and coregistered from the multiphase contrast-enhanced CT (CECT) scans. CECT images of the renal masses were used as inputs to a CECT texture analysis panel comprising 31 texture metrics derived with six texture methods. Stepwise logistic regression analysis was used to select the best predictor from each of the texture methods and performance was assessed.	Entropy, entropy of fast-Fourier transform magnitude, mean, uniformity, information measure of correlation 2, and sum of averages were among the texture predictors aiding renal mass subtyping. The overall CECT-based tumor texture model was accurate for differentiating benign from malignant solid enhancing lipid-poor renal masses.	Insufficient disclosure of the results: AUC given, but no sensitivity or specificity.
**Varghese (3) [92]**	**2018**	Differentiating solid, non-macroscopic fat containing, enhancing renal masses using Fourier analysis of multiphase CT."	156	CT	Histology (surgical)	Differentiation of malignant and benign renal masses (various subtypes): Whole lesions were manually segmented using Synapse 3D (Fujifilm, CT) and co-registered from the multiphase CT acquisitions for each tumor to test the feasibility of two-dimensional fast Fourier transforms (FFT)-based imaging metrics for differentiating solid, non-macroscopic fat containing, enhancing renal masses in contrast-enhanced CT images. Matlab function, FFT2 was used to perform the image to frequency transformation.	FFT-based metrics were different between 1. benign versus malignant renal masses, 2. oncocytoma versus ccRCC, and 3. oncocytoma versus AMLwvf.	Insufficient disclosure of the results: AUC given, but no sensitivity or specificity.
**Xi [97]**	**2020**	Deep Learning to Distinguish Benign from Malignant Renal Lesions Based on Routine MR Imaging."	n.a.	MRI	Histology (surgical)	A deep learning model namely residual convolutional neural network (ResNet) is used to differentiate malignant and benign renal masses on routine MRI-scans. Study population was divided into training, validation and test groups and deep learning was applied. Results were compared to qualitative assessment by a radiologist and a standard radiomics model.	Deep learning methods inherit a high diagnostic accuracy in determining malignancy of renal masses on MR Imaging, even compared to expert or standard radiomics assessment.	
**Yap [101]**	**2018**	Quantitative Contour Analysis as an Image-based Discriminator Between Benign and Malignant Renal Tumors.	150	CT	Histology (surgical)	Differentiation of malignant and benign renal masses by contour assessment on CT images. On the basis of manually segmented tumors 3D models were created using MATLAB software. Morphological data was evaluated in its diagnostic potential. 10 parameters of shape description were included in the analysis.	Morphometry can be applied for discriminating benign and malignant renal masses in terms of convex hull perimeter ration and elliptic compactness. No other tumor shape descriptors proved statistically significant in predicting malignancy.	Insufficient disclosure of the results: No AUC, sensitivity or specificity given, only comparison, only comparison of shape descriptors.

**Zabihollahy [107]**	**2020**	Automated classification of solid renal masses on contrast-enhanced computed tomography images using convolutional neural network with decision fusion	315	CT	Histology (surgical)	Differentiation of benign and malignant RCC in contrast-enhanced CT imaging with the help of deep learning algorithms. Convolutional neural network was combined with decision-fusion testing semi- and fully-automated segmentation and renal tumor evaluation. Thirdly, a 3D model was established. For each model accuracy, precision and recall was measured.	Highest diagnostic accuracy was achieved by semi-automated machine learning models using CNN algorithms.	Insufficient disclosure of the results: AUC given, but no sensitivity or specificity.
**Zabihollahy [109]**	**2020**	Patch-Based Convolutional Neural Network for Differentiation of Cyst From Solid Renal Mass on Contrast-Enhanced Computed Tomography Images.	315	CT	Histology (surgical)	Differentiation of benign renal cysts and solid renal masses by applying automatic convolutional neural network (CNN) to contrast-enhanced CT imaging. Segmentation was performed manually, and patches were labelled according to cystic or solid differentiation. For final diagnosis CNN evaluation was combined with majority voting system algorithms according to detected Hounsfield Units. Training population contained 40 datasets and data augmentation was applied.	Convolutional neural network is a deep learning model applicable to accurate discrimination between cystic and solid renal lesions.	Insufficient disclosure of the results: AUC given, but no sensitivity or specificity.
**Zhou [113]**	**2019**	A Deep Learning-based radiomics model for differentiating benign and malignant renal tumors	192	CT	Histology(surgical)	Differentiation of malignant and benign renal masses combining radiomics with deep learning algorithms. The study population was divided into a training and testing group. 1 Image-level model and 2 patient-level models including 3D-datasets were established for each dataset to evaluate and cross-validate diagnostic accuracy.	Highest diagnostic accuracy for renal mass discrimination was achieved by patient-level deep learning models incorporating 3D data reconstruction.	

Abbreviations: AMLwvf = angiomyolipoma without visible fat, ccRCC = clear-cell renal cell carcinoma, papRCC = papillary renal cell carcinoma, RCC = renal cell carcinoma, chrRCC = chromophobe renal cell carcinoma.

Supplementary Table S5: Studies investigating treatment response assessment using radiomics (n = 6)

Study	Treatment	ROI	Endpoint	Features	Treatment related changes recognized	Treatment related changes not recognized	Total number of included patients
**Antunes 2016 [1]**	sunitinib	Primary tumor	Treatment-related changes in multiple radiomics features after early sunitinib treatment via FLT-PET/MRI	SUV, ADC energy, T2w difference average	2	0	2
**Bharwani 2014 [4]**	sunitinib	Primary tumor	Changes in histogram parameters (ADC and AUC low (proportion of the tumor with ADC values below the 25th percentile of the ADC histograms)) and correlation with OS (baseline (n=26) and treatment related changes in surviving patients (n=20))	whole tumor mean ADC	9	11	20
**Boos 2017 [6]**	TKI (sunitinib n =18, sorafenib n=1)	A measurable soft tissue lesion	Change in CT intensity distribution curves	mean and median lesion attenuation (HU)	11	8	19
**Goh 2011 [29]**	TKI (sunitinib n=26, cedirinib n=6, pazopanib n=4, regorafenib n=3)	metastases	Changes in histogram parameters (entropy and uniformity) and correlation of texture parameters with Time to Progression	Entropy and uniformity	n.a.	n.a.	87
**Haider 2017 [31]**	Sunitinib	A measurable lesion	Correlation of texture parameters with OS and PFS	Entropy following treatment, normalized standard deviation	n.a.	n.a.	87

				prior to and following treatment			
**Mains 2018 [63]**	Various treatments (not specified)	A large artery	Association between OS and PFS with functional CT parameters	Blood volume, blood flow and standardized perfusion	n.a.	n.a.	69

Abbreviations: ST = systemic therapy, ROI = region of interest, FLT-PET/MRI = F 18 fluorothymidine-positron emission tomography/magnetic resonance imaging, SUV = standardized uptake value, ADC = apparent diffusion coefficient, AUC = area under the curve, OS = overall survival, CT = computed tomography, HU = Hounsfield units, PFS = progression free survival.

References

1. Antunes, J., et al., *Radiomics Analysis on FLT-PET/MRI for Characterization of Early Treatment Response in Renal Cell Carcinoma: A Proof-of-Concept Study.* Transl Oncol, 2016. **9**(2): p. 155-162.
2. Bagci, U., et al., *Predicting future morphological changes of lesions from radiotracer uptake in 18F-FDG-PET images.* PLoS One, 2013. **8**(2): p. e57105.
3. Bektas, C.T., et al., *Clear Cell Renal Cell Carcinoma: Machine Learning-Based Quantitative Computed Tomography Texture Analysis for Prediction of Fuhrman Nuclear Grade.* Eur Radiol, 2019. **29**(3): p. 1153-1163.
4. Bharwani, N., et al., *Diffusion-weighted and multiphase contrast-enhanced MRI as surrogate markers of response to neoadjuvant sunitinib in metastatic renal cell carcinoma.* Br J Cancer, 2014. **110**(3): p. 616-24.
5. Bier, G., et al., *Value of computed tomography texture analysis for prediction of perioperative complications during laparoscopic partial nephrectomy in patients with renal cell carcinoma.* PLoS One, 2018. **13**(4): p. e0195270.
6. Boos, J., et al., *CT Intensity Distribution Curve (Histogram) Analysis of Patients Undergoing Antiangiogenic Therapy for Metastatic Renal Cell Carcinoma.* AJR Am J Roentgenol, 2017. **209**(2): p. W85-w92.
7. Catalano, O.A., et al., *Pixel distribution analysis: can it be used to distinguish clear cell carcinomas from angiomyolipomas with minimal fat?* Radiology, 2008. **247**(3): p. 738-46.
8. Chandarana, H., et al., *Histogram analysis of whole-lesion enhancement in differentiating clear cell from papillary subtype of renal cell cancer.* Radiology, 2012. **265**(3): p. 790-8.
9. Chaudhry, H.S., et al., *Histogram analysis of small solid renal masses: differentiating minimal fat angiomyolipoma from renal cell carcinoma.* AJR Am J Roentgenol, 2012. **198**(2): p. 377-83.
10. Chen, F., et al., *Voxel-based whole-lesion enhancement parameters: a study of its clinical value in differentiating clear cell renal cell carcinoma from renal oncocytoma.* Abdom Radiol (NY), 2017. **42**(2): p. 552-560.
11. Chen, F., et al., *Whole lesion quantitative CT evaluation of renal cell carcinoma: differentiation of clear cell from papillary renal cell carcinoma.* Springerplus, 2015. **4**: p. 66.
12. Chen, X., et al., *Reliable gene mutation prediction in clear cell renal cell carcinoma through multi-classifier multi-objective radiogenomics model.* Phys Med Biol, 2018. **63**(21): p. 215008.
13. Coy, H., et al., *Deep learning and radiomics: the utility of Google TensorFlow™ Inception in classifying clear cell renal cell carcinoma and oncocytoma on multiphasic CT.* Abdom Radiol (NY), 2019. **44**(6): p. 2009-2020.
14. Cui, E.M., et al., *Differentiation of renal angiomyolipoma without visible fat from renal cell carcinoma by machine learning based on whole-tumor computed tomography texture features.* Acta Radiol, 2019. **60**(11): p. 1543-1552.
15. Cui, E., et al., *Predicting the ISUP grade of clear cell renal cell carcinoma with multiparametric MR and multiphase CT radiomics.* Eur Radiol, 2020. **30**(5): p. 2912-2921.
16. Deng, Y., et al., *Usefulness of CT texture analysis in differentiating benign and malignant renal tumours.* Clin Radiol, 2020. **75**(2): p. 108-115.
17. Deng, Y., et al., *CT texture analysis in the differentiation of major renal cell carcinoma subtypes and correlation with Fuhrman grade.* Eur Radiol, 2019. **29**(12): p. 6922-6929.
18. Ding, J., et al., *CT-based radiomic model predicts high grade of clear cell renal cell carcinoma.* Eur J Radiol, 2018. **103**: p. 51-56.

19. Doshi, A.M., et al., *Use of MRI in Differentiation of Papillary Renal Cell Carcinoma Subtypes: Qualitative and Quantitative Analysis.* AJR Am J Roentgenol, 2016. **206**(3): p. 566-72.
20. Dwivedi, D.K., et al., *Development of a Patient-specific Tumor Mold Using Magnetic Resonance Imaging and 3-Dimensional Printing Technology for Targeted Tissue Procurement and Radiomics Analysis of Renal Masses.* Urology, 2018. **112**: p. 209-214.
21. Erdim, C., et al., *Prediction of Benign and Malignant Solid Renal Masses: Machine Learning-Based CT Texture Analysis.* Acad Radiol, 2020.
22. Feng, Z., et al., *Machine learning-based quantitative texture analysis of CT images of small renal masses: Differentiation of angiomyolipoma without visible fat from renal cell carcinoma.* Eur Radiol, 2018. **28**(4): p. 1625-1633.
23. Feng, Z., et al., *CT texture analysis: a potential tool for predicting the Fuhrman grade of clear-cell renal carcinoma.* Cancer Imaging, 2019. **19**(1): p. 6.
24. Feng, Z., et al., *Identifying BAP1 Mutations in Clear-Cell Renal Cell Carcinoma by CT Radiomics: Preliminary Findings.* Front Oncol, 2020. **10**: p. 279.
25. Gaing, B., et al., *Subtype differentiation of renal tumors using voxel-based histogram analysis of intravoxel incoherent motion parameters.* Invest Radiol, 2015. **50**(3): p. 144-52.
26. Ghosh, P., et al., *Imaging-genomic pipeline for identifying gene mutations using three-dimensional intra-tumor heterogeneity features.* J Med Imaging (Bellingham), 2015. **2**(4): p. 041009.
27. Gill, T.S., et al., *Juxtatumoral perinephric fat analysis in clear cell renal cell carcinoma.* Abdom Radiol (NY), 2019. **44**(4): p. 1470-1480.
28. Gillingham, N., et al., *Bosniak IIF and III Renal Cysts: Can Apparent Diffusion Coefficient-Derived Texture Features Discriminate Between Malignant and Benign IIF and III Cysts?* J Comput Assist Tomogr, 2019. **43**(3): p. 485-492.
29. Goh, V., et al., *Assessment of response to tyrosine kinase inhibitors in metastatic renal cell cancer: CT texture as a predictive biomarker.* Radiology, 2011. **261**(1): p. 165-71.
30. Goyal, A., et al., *Role of MR texture analysis in histological subtyping and grading of renal cell carcinoma: a preliminary study.* Abdom Radiol (NY), 2019. **44**(10): p. 3336-3349.
31. Haider, M.A., et al., *CT texture analysis: a potential tool for prediction of survival in patients with metastatic clear cell carcinoma treated with sunitinib.* Cancer Imaging, 2017. **17**(1): p. 4.
32. Han, S., S.I. Hwang, and H.J. Lee, *The Classification of Renal Cancer in 3-Phase CT Images Using a Deep Learning Method.* Journal of Digital Imaging, 2019. **32**(4): p. 638-643.
33. He, X., et al., *Grading of Clear Cell Renal Cell Carcinomas by Using Machine Learning Based on Artificial Neural Networks and Radiomic Signatures Extracted From Multidetector Computed Tomography Images.* Acad Radiol, 2020. **27**(2): p. 157-168.
34. He, X., et al., *Predictive models composed by radiomic features extracted from multi-detector computed tomography images for predicting low- and high- grade clear cell renal cell carcinoma: A STARD-compliant article.* Medicine (Baltimore), 2019. **98**(2): p. e13957.
35. Hoang, U.N., et al., *Assessment of multiphasic contrast-enhanced MR textures in differentiating small renal mass subtypes.* Abdom Radiol (NY), 2018. **43**(12): p. 3400-3409.
36. Hodgdon, T., et al., *Can Quantitative CT Texture Analysis be Used to Differentiate Fat-poor Renal Angiomyolipoma from Renal Cell Carcinoma on Unenhanced CT Images?* Radiology, 2015. **276**(3): p. 787-96.
37. Huhdanpaa, H., et al., *CT prediction of the Fuhrman grade of clear cell renal cell carcinoma (RCC): towards the development of computer-assisted diagnostic method.* Abdom Imaging, 2015. **40**(8): p. 3168-74.

38. Khene, Z.E., et al., *Role of quantitative computed tomography texture analysis in the prediction of adherent perinephric fat.* World J Urol, 2018. **36**(10): p. 1635-1642.
39. Kierans, A.S., et al., *Textural differences in apparent diffusion coefficient between low- and high-stage clear cell renal cell carcinoma.* AJR Am J Roentgenol, 2014. **203**(6): p. W637-44.
40. Kim, J.Y., et al., *CT histogram analysis: differentiation of angiomyolipoma without visible fat from renal cell carcinoma at CT imaging.* Radiology, 2008. **246**(2): p. 472-9.
41. Kim, N.Y., et al., *Utility of CT Texture Analysis in Differentiating Low-Attenuation Renal Cell Carcinoma From Cysts: A Bi-Institutional Retrospective Study.* AJR Am J Roentgenol, 2019. **213**(6): p. 1259-1266.
42. Kocak, B., et al., *Machine learning-based unenhanced CT texture analysis for predicting BAP1 mutation status of clear cell renal cell carcinomas.* Acta Radiol, 2019: p. 284185119881742.
43. Kocak, B., et al., *Influence of segmentation margin on machine learning-based high-dimensional quantitative CT texture analysis: a reproducibility study on renal clear cell carcinomas.* Eur Radiol, 2019. **29**(9): p. 4765-4775.
44. Kocak, B., et al., *Unenhanced CT Texture Analysis of Clear Cell Renal Cell Carcinomas: A Machine Learning-Based Study for Predicting Histopathologic Nuclear Grade.* AJR Am J Roentgenol, 2019: p. W1-w8.
45. Kocak, B., et al., *Radiogenomics in Clear Cell Renal Cell Carcinoma: Machine Learning-Based High-Dimensional Quantitative CT Texture Analysis in Predicting PBRM1 Mutation Status.* AJR Am J Roentgenol, 2019. **212**(3): p. W55-w63.
46. Kocak, B., et al., *Reliability of Single-Slice-Based 2D CT Texture Analysis of Renal Masses: Influence of Intra- and Interobserver Manual Segmentation Variability on Radiomic Feature Reproducibility.* AJR Am J Roentgenol, 2019. **213**(2): p. 377-383.
47. Kocak, B., et al., *Textural differences between renal cell carcinoma subtypes: Machine learning-based quantitative computed tomography texture analysis with independent external validation.* Eur J Radiol, 2018. **107**: p. 149-157.
48. Kunapuli, G., et al., *A Decision-Support Tool for Renal Mass Classification.* J Digit Imaging, 2018. **31**(6): p. 929-939.
49. Lee, H., et al., *Deep feature classification of angiomyolipoma without visible fat and renal cell carcinoma in abdominal contrast-enhanced CT images with texture image patches and hand-crafted feature concatenation.* Med Phys, 2018. **45**(4): p. 1550-1561.
50. Lee, H.S., et al., *Differentiation of fat-poor angiomyolipoma from clear cell renal cell carcinoma in contrast-enhanced MDCT images using quantitative feature classification.* Med Phys, 2017. **44**(7): p. 3604-3614.
51. Lee, H.W., et al., *Integrative Radiogenomics Approach for Risk Assessment of Post-Operative Metastasis in Pathological T1 Renal Cell Carcinoma: A Pilot Retrospective Cohort Study.* Cancers (Basel), 2020. **12**(4).
52. Leng, S., et al., *Subjective and objective heterogeneity scores for differentiating small renal masses using contrast-enhanced CT.* Abdom Radiol (NY), 2017. **42**(5): p. 1485-1492.
53. Li, A.Q., et al., *Subtype Differentiation of Small (<= 4 cm) Solid Renal Mass Using Volumetric Histogram Analysis of DWI at 3-T MRI.* American Journal of Roentgenology, 2018. **211**(3): p. 614-623.
54. Li, H., et al., *Whole-Tumor Quantitative Apparent Diffusion Coefficient Histogram and Texture Analysis to Differentiation of Minimal Fat Angiomyolipoma from Clear Cell Renal Cell Carcinoma.* Acad Radiol, 2019. **26**(5): p. 632-639.
55. Li, Y., et al., *Value of radiomics in differential diagnosis of chromophobe renal cell carcinoma and renal oncocytoma.* Abdom Radiol (NY), 2019.

56. Li, Z.C., et al., *Differentiation of clear cell and non-clear cell renal cell carcinomas by all-relevant radiomics features from multiphase CT: a VHL mutation perspective.* Eur Radiol, 2019. **29**(8): p. 3996-4007.
57. Lin, F., et al., *CT-based machine learning model to predict the Fuhrman nuclear grade of clear cell renal cell carcinoma.* Abdom Radiol (NY), 2019. **44**(7): p. 2528-2534.
58. Linguraru, M.G., et al., *Computer-aided renal cancer quantification and classification from contrast-enhanced CT via histograms of curvature-related features.* Conf Proc IEEE Eng Med Biol Soc, 2009. **2009**: p. 6679-82.
59. Linguraru, M.G., et al., *Automated noninvasive classification of renal cancer on multiphase CT.* Med Phys, 2011. **38**(10): p. 5738-46.
60. Liu, G.S., et al., *The value of CT image-based texture analysis for differentiating renal primary undifferentiated pleomorphic sarcoma from three subtypes of renal cell carcinoma.* International Journal of Clinical and Experimental Medicine, 2017. **10**(9): p. 13526-13533.
61. Lubner, M.G., et al., *CT Textural Analysis of Large Primary Renal Cell Carcinomas: Pretreatment Tumor Heterogeneity Correlates With Histologic Findings and Clinical Outcomes.* AJR Am J Roentgenol, 2016. **207**(1): p. 96-105.
62. Ma, Y., et al., *Can whole-tumor radiomics-based CT analysis better differentiate fat-poor angiomyolipoma from clear cell renal cell caricinoma: compared with conventional CT analysis?* Abdom Radiol (NY), 2020.
63. Mains, J.R., et al., *Use of patient outcome endpoints to identify the best functional CT imaging parameters in metastatic renal cell carcinoma patients.* Br J Radiol, 2018. **91**(1082): p. 20160795.
64. Marigliano, C., et al., *Radiogenomics in Clear Cell Renal Cell Carcinoma: Correlations Between Advanced CT Imaging (Texture Analysis) and MicroRNAs Expression.* Technol Cancer Res Treat, 2019. **18**: p. 1533033819878458.
65. Nazari, M., et al., *Noninvasive Fuhrman grading of clear cell renal cell carcinoma using computed tomography radiomic features and machine learning.* Radiol Med, 2020.
66. Nie, P., et al., *A CT-based radiomics nomogram for differentiation of renal angiomyolipoma without visible fat from homogeneous clear cell renal cell carcinoma.* Eur Radiol, 2020. **30**(2): p. 1274-1284.
67. Paschall, A.K., et al., *Differentiating papillary type I RCC from clear cell RCC and oncocytoma: application of whole-lesion volumetric ADC measurement.* Abdom Radiol (NY), 2018. **43**(9): p. 2424-2430.
68. Picard, M., et al., *Combined Qualitative and Quantitative Assessment of Low-Attenuation Renal Lesions Improves Identification of Renal Malignancy on Noncontrast Computed Tomography*. J Comput Assist Tomogr, 2019. **43**(6): p. 852-856.
69. Purkayastha, S., et al., *Differentiation of low and high grade renal cell carcinoma on routine MRI with an externally validated automatic machine learning algorithm.* Sci Rep, 2020. **10**(1): p. 19503.
70. Raman, S.P., et al., *CT texture analysis of renal masses: pilot study using random forest classification for prediction of pathology.* Acad Radiol, 2014. **21**(12): p. 1587-96.
71. Ramesh, G.e.a., *Assessment of Primary Solid Renal Mass using Texture Analysis of CT Images of Kidney by Active Contour Method: A Novel Method*. Journal of Clinical and Diagnostic Research, 2018. **12**(7): p. TC05 - TC09.
72. Reynolds, H.M., et al., *Diffusion weighted and dynamic contrast enhanced MRI as an imaging biomarker for stereotactic ablative body radiotherapy (SABR) of primary renal cell carcinoma.* PLoS One, 2018. **13**(8): p. e0202387.
73. Said, D., et al., *Characterization of solid renal neoplasms using MRI-based quantitative radiomics features.* Abdom Radiol (NY), 2020.

74. Sasaguri, K., et al., *Small (< 4 cm) Renal Mass: Differentiation of Oncocytoma From Renal Cell Carcinoma on Biphasic Contrast-Enhanced CT.* AJR Am J Roentgenol, 2015. **205**(5): p. 999-1007.
75. Schieda, N., et al., *Diagnostic Accuracy of Unenhanced CT Analysis to Differentiate Low-Grade From High-Grade Chromophobe Renal Cell Carcinoma.* AJR Am J Roentgenol, 2018. **210**(5): p. 1079-1087.
76. Schieda, N., et al., *Diagnosis of Sarcomatoid Renal Cell Carcinoma With CT: Evaluation by Qualitative Imaging Features and Texture Analysis.* AJR Am J Roentgenol, 2015. **204**(5): p. 1013-23.
77. Scrima, A.T., et al., *Texture analysis of small renal cell carcinomas at MDCT for predicting relevant histologic and protein biomarkers.* Abdom Radiol (NY), 2019. **44**(6): p. 1999-2008.
78. Simpfendorfer, C., et al., *Angiomyolipoma with minimal fat on MDCT: can counts of negative-attenuation pixels aid diagnosis?* AJR Am J Roentgenol, 2009. **192**(2): p. 438-43.
79. Shu, J., et al., *Clear cell renal cell carcinoma: CT-based radiomics features for the prediction of Fuhrman grade.* Eur J Radiol, 2018. **109**: p. 8-12.
80. Shu, J., et al., *Clear cell renal cell carcinoma: Machine learning-based computed tomography radiomics analysis for the prediction of WHO/ISUP grade.* Eur J Radiol, 2019. **121**: p. 108738.
81. Soma, T., et al., *Potential for computer-aided diagnosis using a convolutional neural network algorithm to diagnose fat-poor angiomyolipoma in enhanced computed tomography and T2-weighted magnetic resonance imaging.* Int J Urol, 2018. **25**(11): p. 978-979.
82. Stanzione, A., et al., *MRI Radiomics for the Prediction of Fuhrman Grade in Clear Cell Renal Cell Carcinoma: a Machine Learning Exploratory Study.* J Digit Imaging, 2020.
83. Sun, X., et al., *Prediction of ISUP grading of clear cell renal cell carcinoma using support vector machine model based on CT images.* Medicine (Baltimore), 2019. **98**(14): p. e15022.
84. Sun, X.Y., et al., *Radiologic-Radiomic Machine Learning Models for Differentiation of Benign and Malignant Solid Renal Masses: Comparison With Expert-Level Radiologists.* AJR Am J Roentgenol, 2020. **214**(1): p. W44-w54.
85. Takahashi, N., et al., *Small (< 4 cm) Renal Masses: Differentiation of Angiomyolipoma Without Visible Fat From Renal Cell Carcinoma Using Unenhanced and Contrast-Enhanced CT.* AJR Am J Roentgenol, 2015. **205**(6): p. 1194-202.
86. Takahashi, N., et al., *CT negative attenuation pixel distribution and texture analysis for detection of fat in small angiomyolipoma on unenhanced CT.* Abdom Radiol (NY), 2016. **41**(6): p. 1142-51.
87. Tanaka, H., et al., *Diffusion-weighted magnetic resonance imaging in the differentiation of angiomyolipoma with minimal fat from clear cell renal cell carcinoma.* Int J Urol, 2011. **18**(10): p. 727-30.
88. Tanaka, T., et al., *Differentiation of Small (≤ 4 cm) Renal Masses on Multiphase Contrast-Enhanced CT by Deep Learning.* AJR Am J Roentgenol, 2020. **214**(3): p. 605-612.
89. Tang, Z., et al., *Quantitative Analysis of Multiphase Contrast-Enhanced CT Images: A Pilot Study of Preoperative Prediction of Fat-Poor Angiomyolipoma and Renal Cell Carcinoma.* AJR Am J Roentgenol, 2020. **214**(2): p. 370-382.
90. Uhlig, J., et al., *Discriminating malignant and benign clinical T1 renal masses on computed tomography: A pragmatic radiomics and machine learning approach.* Medicine (Baltimore), 2020. **99**(16): p. e19725.
91. Varghese, B.A., et al., *Differentiation of Predominantly Solid Enhancing Lipid-Poor Renal Cell Masses by Use of Contrast-Enhanced CT: Evaluating the Role of Texture in Tumor Subtyping.* AJR Am J Roentgenol, 2018. **211**(6): p. W288-w296.

92. Varghese, B.A., et al., *Differentiating solid, non-macroscopic fat containing, enhancing renal masses using fast Fourier transform analysis of multiphase CT.* Br J Radiol, 2018. **91**(1089): p. 20170789.
93. Vendrami, C.L., et al., *Differentiation of Papillary Renal Cell Carcinoma Subtypes on MRI: Qualitative and Texture Analysis.* AJR Am J Roentgenol, 2018. **211**(6): p. 1234-1245.
94. Wang, H.Y., et al., *Dynamic Contrast-enhanced MR Imaging in Renal Cell Carcinoma: Reproducibility of Histogram Analysis on Pharmacokinetic Parameters.* Sci Rep, 2016. **6**: p. 29146.
95. Wang, W., et al., *Differentiation of renal cell carcinoma subtypes through MRI-based radiomics analysis.* Eur Radiol, 2020.
96. Xi, Y., et al., *Statistical clustering of parametric maps from dynamic contrast enhanced MRI and an associated decision tree model for non-invasive tumour grading of T1b solid clear cell renal cell carcinoma.* Eur Radiol, 2018. **28**(1): p. 124-132.
97. Xi, I.L., et al., *Deep Learning to Distinguish Benign from Malignant Renal Lesions Based on Routine MR Imaging.* Clin Cancer Res, 2020. **26**(8): p. 1944-1952.
98. Yan, L., et al., *Angiomyolipoma with minimal fat: differentiation from clear cell renal cell carcinoma and papillary renal cell carcinoma by texture analysis on CT images.* Acad Radiol, 2015. **22**(9): p. 1115-21.
99. Yang, G., et al., *Contrast-Enhanced CT Texture Analysis for Distinguishing Fat-Poor Renal Angiomyolipoma From Chromophobe Renal Cell Carcinoma.* Mol Imaging, 2019. **18**: p. 1536012119883161.
100. Yang, R., et al., *Radiomics of small renal masses on multiphasic CT: accuracy of machine learning-based classification models for the differentiation of renal cell carcinoma and angiomyolipoma without visible fat.* Eur Radiol, 2020. **30**(2): p. 1254-1263.
101. Yap, F.Y., et al., *Quantitative Contour Analysis as an Image-based Discriminator Between Benign and Malignant Renal Tumors.* Urology, 2018. **114**: p. 121-127.
102. Yaşar, S., et al., *Using texture analysis as a predictive factor of subtype, grade and stage of renal cell carcinoma.* Abdom Radiol (NY), 2020.
103. Yin, Q., et al., *Integrative radiomics expression predicts molecular subtypes of primary clear cell renal cell carcinoma.* Clin Radiol, 2018. **73**(9): p. 782-791.
104. Yin, Q., et al., *Associations between Tumor Vascularity, Vascular Endothelial Growth Factor Expression and PET/MRI Radiomic Signatures in Primary Clear-Cell-Renal-Cell-Carcinoma: Proof-of-Concept Study.* Sci Rep, 2017. **7**: p. 43356.
105. You, M.W., N. Kim, and H.J. Choi, *The value of quantitative CT texture analysis in differentiation of angiomyolipoma without visible fat from clear cell renal cell carcinoma on four-phase contrast-enhanced CT images.* Clin Radiol, 2019. **74**(7): p. 547-554.
106. Yu, H., et al., *Texture analysis as a radiomic marker for differentiating renal tumors.* Abdom Radiol (NY), 2017. **42**(10): p. 2470-2478.
107. Zabihollahy, F., et al., *Automated classification of solid renal masses on contrast-enhanced computed tomography images using convolutional neural network with decision fusion.* Eur Radiol, 2020.
108. Zabihollahy, F., et al., *Ensemble U-Net-Based Method for Fully Automated Detection and Segmentation of Renal Masses on Computed Tomography Images.* Med Phys, 2020.
109. Zabihollahy, F., N. Schieda, and E. Ukwatta, *Patch-Based Convolutional Neural Network for Differentiation of Cyst From Solid Renal Mass on Contrast-Enhanced Computed Tomography Images.* Ieee Access, 2020. **8**: p. 8595-8602.
110. Zhang, G.M., et al., *Can quantitative CT texture analysis be used to differentiate subtypes of renal cell carcinoma?* Clin Radiol, 2019. **74**(4): p. 287-294.

111. Zhang, H., et al., *[A radiomic approach to differential diagnosis of renal cell carcinoma in patients with hydronephrosis and renal calculi]*. Nan Fang Yi Ke Da Xue Xue Bao, 2019. **39**(5): p. 547-553.
112. Zhang, Y.D., et al., *Comparison of Utility of Histogram Apparent Diffusion Coefficient and R2* for Differentiation of Low-Grade From High-Grade Clear Cell Renal Cell Carcinoma.* AJR Am J Roentgenol, 2015. **205**(2): p. W193-201.
113. Zhou, L., et al., *A Deep Learning-Based Radiomics Model for Differentiating Benign and Malignant Renal Tumors.* Transl Oncol, 2019. **12**(2): p. 292-300.
114. Li, A., et al., *Subtype Differentiation of Small (≤ 4 cm) Solid Renal Mass Using Volumetric Histogram Analysis of DWI at 3-T MRI.* AJR Am J Roentgenol, 2018. **211**(3): p. 614-623.
